# Human ventromedial prefrontal lesions alter incentivisation by reward

**DOI:** 10.1016/j.cortex.2016.01.005

**Published:** 2016-03

**Authors:** Sanjay G. Manohar, Masud Husain

**Affiliations:** a Nuffield Dept of Clinical Neurosciences, John Radcliffe Hospital, University of Oxford, UK; b Dept of Experimental Psychology, University of Oxford, UK

**Keywords:** Lesion, Motivation, Reward, Subarachnoid haemorrhage, Ventromedial prefrontal cortex

## Abstract

Although medial frontal brain regions are implicated in valuation of rewards, evidence from focal lesions to these areas is scant, with many conflicting results regarding motivation and affect, and no human studies specifically examining incentivisation by reward. Here, 19 patients with isolated, focal damage in ventral and medial prefrontal cortex were selected from a database of 453 individuals with subarachnoid haemorrhage. Using a speeded saccadic task based on the oculomotor capture paradigm, we manipulated the maximum reward available on each trial using an auditory incentive cue. Modulation of behaviour by motivation permitted quantification of reward sensitivity. At the group level, medial frontal damage was overall associated with significantly reduced effects of reward on invigorating saccadic velocity and autonomic (pupil) responses compared to age-matched, healthy controls. Crucially, however, some individuals instead showed abnormally strong incentivisation effects for vigour. *Increased* sensitivity to rewards within the lesion group correlated with damage in subgenual ventromedial prefrontal cortex (vmPFC) areas, which have recently become the target for deep brain stimulation (DBS) in depression. Lesion correlations with clinical apathy suggested that the apathy associated with prefrontal damage is in fact reduced by damage at those coordinates. *Reduced* reward sensitivity showed a trend to correlate with damage near nucleus accumbens. Lesions did not, on the other hand, influence reward sensitivity of cognitive control, as measured by distractibility. Thus, although medial frontal lesions may generally reduce reward sensitivity, damage to key subregions paradoxically protect from this effect.

## 1 Introduction

In recent years, investigations of cortical reward value representations have focused heavily on the role of ventromedial prefrontal cortex (vmPFC), sometimes also referred to as *medial* orbitofrontal cortex (Bartra et al., 2013, Hayes et al., 2014; reviewed in Clithero and Rangel, 2014, Levy and Glimcher, 2012, Ruff and Fehr, 2014). But although vmPFC has been extensively implicated in computing reward value in human functional imaging studies, some investigators have contested this interpretation (O'Doherty, 2014, Stalnaker et al., 2015). Could the observed reward-related activations instead indicate a role in *regulating* reward signals – for example, in the basal ganglia – as a function of context? To date, animal evidence can be interpreted as weighing in favour of vmPFC playing a regulatory role, rather than its necessity for value-guided behaviour *per se* (Jo and Mizumori, 2015, Moorman and Aston-Jones, 2015, Rudebeck et al., 2013, Schoenbaum et al., 2011). These two viewpoints make differing predictions regarding the effect of lesions. If vmPFC is responsible for computing value, then damage to this region might be expected to reduce the effect of reward on motivated behaviour. On the other hand, if its role were regulatory or modulatory, then damage to this region might paradoxically potentiate some of reward's direct effects.

Malfunctioning of the brain's value computation system has been proposed to underlie two distinct but related syndromes: depression and apathy (Alguacil and González-Martín, 2015, Eshel and Roiser, 2010, Hall et al., 2014, Perry and Kramer, 2015, Rochat et al., 2013, Sinha et al., 2013, Whitton et al., 2015). These behavioural conditions, which occur frequently across a range of brain disorders, have been characterised either as blunted reward sensitivity, or aberrant regulation of reward value (Cipriani et al., 2014, Foussias et al., 2014, Hellmann-Regen et al., 2013, Marin and Wilkosz, 2005). Intriguingly, neuroimaging studies have highlighted abnormal vmPFC activity in both these disorders (Alexopoulos et al., 2013, Drevets et al., 2008, Koenigs and Grafman, 2009, Ubl et al., 2015), and some investigations have even reported that major depression can be successfully alleviated by surgical lesions or deep brain stimulation (DBS) of posterior vmPFC white matter (Bridges et al., 1994, Johansen-Berg et al., 2008, Mayberg et al., 2005, Moreines et al., 2014, Schlaepfer et al., 2007). Taken together, these findings suggest that inappropriate or dysregulated control over reward could characterise affective or motivational disorders. Establishing a link between motivational disorders and human vmPFC damage could therefore provide stronger causal evidence for this region's role.

Studies on human focal lesions involving vmPFC would provide an ideal opportunity to test the role of this region in reward processing. However, focal damage to this region of the brain is relatively uncommon, and those studies that have been conducted have often been based on small numbers of participants. Moreover, reported effects following lesions are heterogeneous and often seemingly conflicting. For example, both apathy as well as impulsivity have been documented (Berlin et al., 2004, Jouvent et al., 2011, Lhermitte, 1986); while blunted affect and emotional lability are frequent (Angrilli et al., 1999, Beer et al., 2003, Ghaffar et al., 2008, Kim and Choi-Kwon, 2000). Furthermore, different studies have suggested either a predisposition to or even protection from depression (Ellenbogen et al., 2005, Kim and Choi-Kwon, 2000, Koenigs and Grafman, 2009, Koenigs et al., 2008, MacFall et al., 2001). From this evidence it is difficult to conclude that lesions to human vmPFC influence reward processing, or impact on motivation. It is possible that motivation in different aspects of behaviour may be differentially affected. Importantly, the question remains open as to whether reward sensitivity would be blunted or increased by damage to this region.

To better characterise effects of lesions, cognitive tasks that attempt to tap specific processes have been employed, e.g., to demonstrate disturbed decision-making following vmPFC lesions (Fellows and Farah, 2005, Gläscher et al., 2012, Levens et al., 2014), though even these have been inconsistent (Manes et al., 2002). Specifically, vmPFC lesions can lead to suboptimal or higher betting in risk-related decisions (Clark et al., 2008, Levens et al., 2014, Studer et al., 2015), coupled with altered autonomic anticipatory responses (Bechara, Damasio, Tranel, & Damasio, 2005). vmPFC patients also exhibit altered reversal learning of stimulus-reward associations (Fellows and Farah, 2003, Hornak et al., 2004, Tsuchida et al., 2010). All these might be consequences of a more pervasive disorder of *evaluation*, as manifest by abnormal and self-inconsistent preferences (Fellows and Farah, 2007, Koenigs and Tranel, 2008). But surprisingly, to the best of our knowledge, there is no study that has directly examined the effect of vmPFC lesions on *incentivisation by reward value* in humans.

Here, our aim was to test the specific role of vmPFC in using value to incentivise action. To do this, we adapted the oculomotor capture task, which has previously provided detailed insights into the automatic effects of reward (Anderson and Yantis, 2012, Hickey et al., 2011, Jazbec et al., 2006, Le Pelley et al., 2015). We used this paradigm in patients with focal damage in the anterior cerebral artery (ACA) territory, following subarachnoid haemorrhage. The task is a simplified variant of the oculomotor capture paradigm (Theeuwes et al., 1998, Van der Stigchel et al., 2012), in which participants have to exert a degree of cognitive control. Similar to an anti-saccade task, participants must look away from a visually salient onset. Crucially, to probe how *motivation by reward incentives* influences behaviour, we varied the amount of money that could be won for each saccade, on a trial-by-trial basis, using an auditory precue. Monetary incentive cues have recently been shown to modulate the velocity of saccades on this task (Manohar et al., 2015). In addition, we assessed autonomic responses to reward on offer by measuring pupillary dilatation. Motivational effects of reward were quantified by saccadic velocity (response vigour), pupillary dilatation (autonomic response) and oculomotor capture (cognitive control) as a function of different reward values. We predicted that vmPFC lesions might alter the effect of reward on these measures.

Our aim here was not to define all brain regions involved in processing reward but to investigate specifically whether medial prefrontal cortical lesions have an impact on reward sensitivity. We used hypothesis-based, region of interest (ROI) predictions as well as whole brain voxel-based lesion mapping specifically to probe regions *within* medial PFC, which when lesioned, lead to alterations in reward sensitivity.

## 2 Material and methods

### 2.1 Participants

**Fig. 1:**
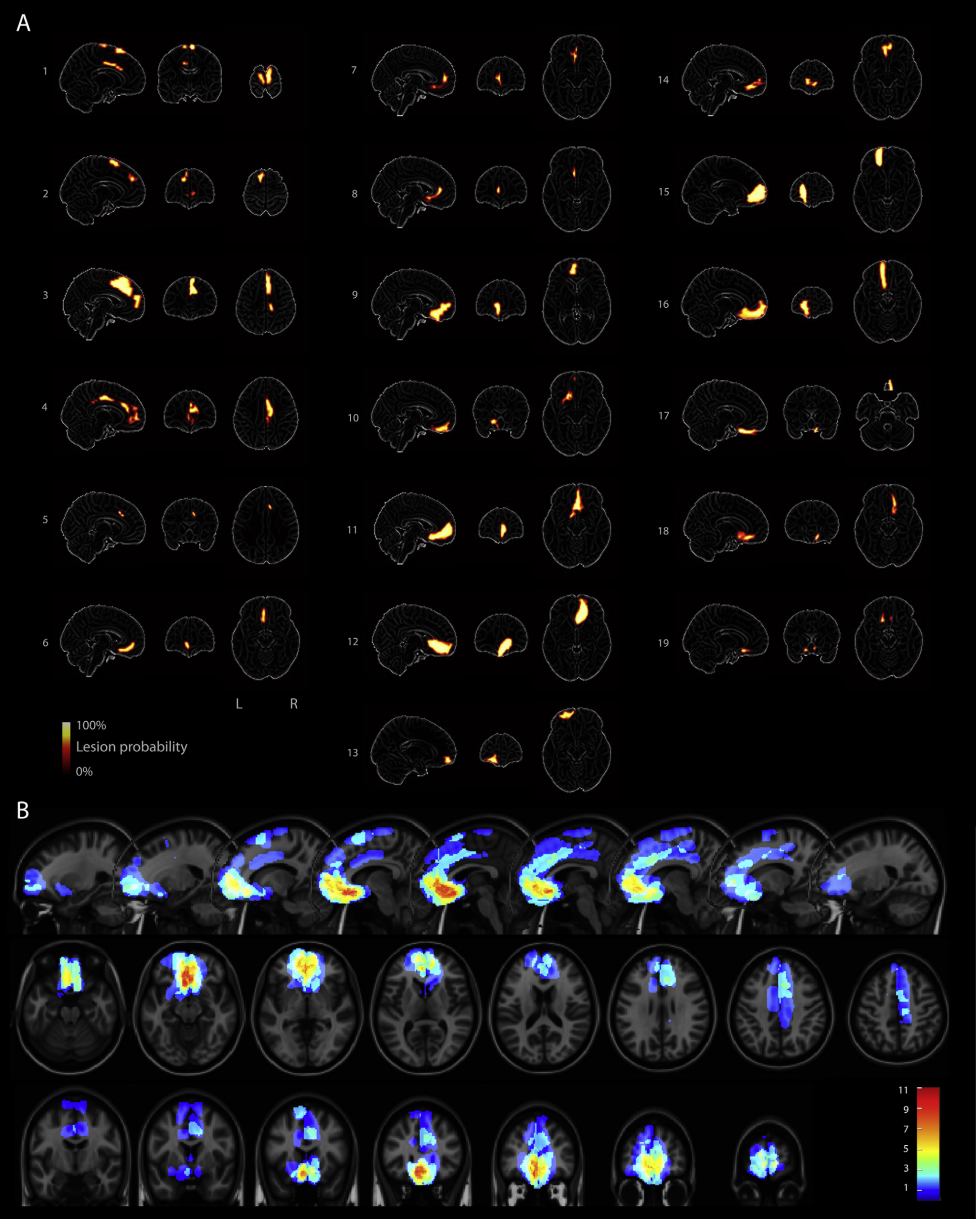
**Individual lesions and overlap map of lesions of 19 patients included in the analysis**. Nineteen patients with focal lesions in medial prefrontal cortex were tested. Patients had suffered subarachnoid haemorrhage between two and five years previously, with consequent anterior cerebral artery territory. Lesions were manually traced onto the MRI scans (CT in one patient). The traced volumetric lesion masks were normalised and smoothly interpolated onto the MNI152 template using cost-function mapping. **A)** Individual patients' lesions. The slice with the greatest number of lesioned voxels, in each plane, is shown over the template. There were no cortical lesions outside of medial frontal areas, but some patients had involvement of subcortical white matter tracts, basal forebrain and anterior striatum. **B)** Overlap map of all patients. Colours indicate how many patients had lesions involving each voxel.

A database of 453 patients who suffered subarachnoid haemorrhages between 2007 and 2012 was screened. Only 22 were found to have isolated focal infarcts in the ACA territory as a consequence of subarachnoid haemorrhages from ACA aneurysms, and were alive and capable of being tested (Fig. 1A). All were tested in a chronic state, 2–5 years after the event. The mean age of our study group was 49.6 years, std. 10.8 years (Table 1). All cases had highly selective lesions involving medial frontal cortex, but with no physical neurological signs, consistent with previous reports (Helbok et al., 2011), except one patient who had downbeat nystagmus which was mild enough to permit eye tracking.

One individual was on olanzapine (case 1) and another on citalopram 10 mg for depression (case 5). None of the others were on psychotropic medication or anticonvulsants. 32 healthy control participants were recruited by advert (mean age 50.6 years). They had no neurological or psychiatric illness and normal or corrected-to-normal vision. All participants gave written consent to participate and all procedures were approved by the local Research Ethics Committee.

Of the 22 patients tested, one had severe fatigue and dropped out; in one case eye movements were difficult to record; and one patient was subsequently found to have a lesion extending into the temporal lobe, so was excluded. Thus eye movement data were available for 19 patients. They were assessed with the Hospital Anxiety and Depression Scale (HADS, Zigmond & Snaith, 1983) and the Lille Apathy Rating Scale (LARS, Sockeel et al., 2006).

Seventeen cases had MR imaging (11 with 2 mm isotropic T1 MRI and 6 with non-isotropic 2 × 2 × 5 mm T1 images). One had only FLAIR images while one case had only a CT scan (these latter two patients had metal surgical clips and an implantable defibrillator). Each patient's lesion was traced manually onto their brain scan by a neurologist (SGM), using FSL (http://fsl.fmrib.ox.ac.uk/fsl). Scans and lesion masks were registered to the MNI152 template. Volumetric T1 scans were registered using SPM8 and the cost-function masking toolbox (Rorden, Karnath, & Bonilha, 2007). Other MRI scans were resampled and registered using FLIRT with trilinear interpolation; linear registration reduces the chance of misalignment due to the lesions (Brett et al., 2001, Crinion et al., 2007). All lesion maps were convolved with a 1 mm full-width-half-maximum Gaussian kernel. The mean lesion volume was 5.7 cm^3^ (SD 5.6), and ranged from .1 to 19.1 cm^3^. An overlap map was constructed by counting in each voxel the number of patients who had a >10% degree of lesion (Fig. 1B).

### 2.2 Task

**Fig. 2:**
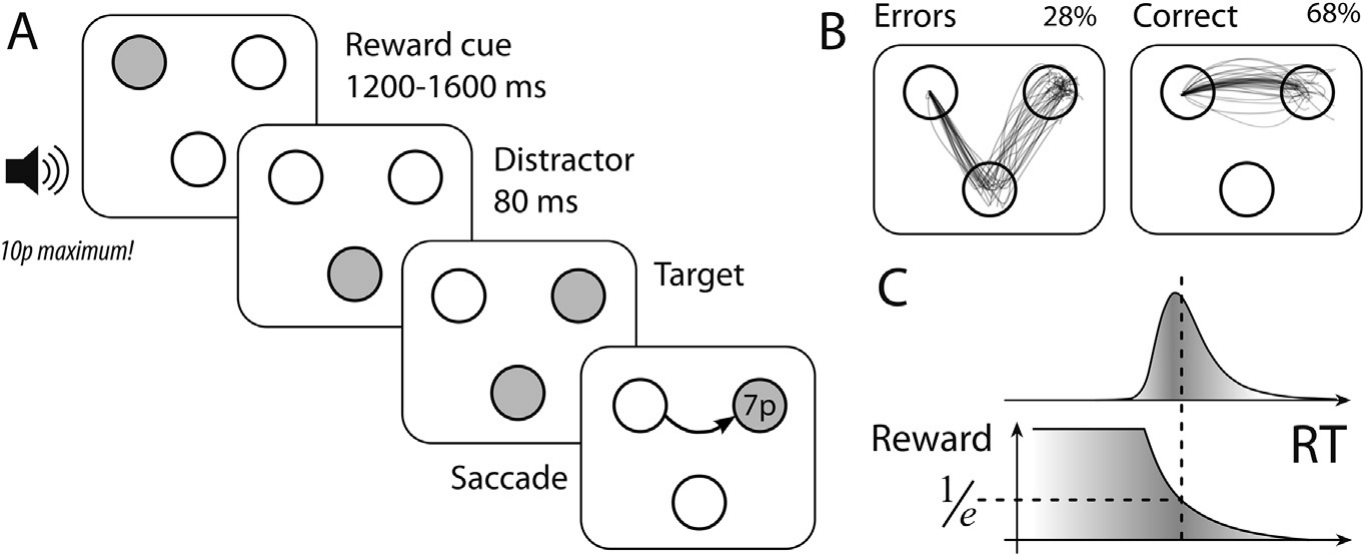
**Monetary incentive saccadic distractor task. A)** Three equidistant discs were dimly illuminated. At the start of each trial, participants had to fixate one disc which was brightened. A recorded voice gave an auditory reward cue (either “0p maximum”, “10p maximum” or “50p maximum”) which indicated the amount of money that could be won if subjects were accurate and fast on that trial. After a variable foreperiod, the other two discs were illuminated asynchronously, with a delay of 80 msec. Participants were required to hold fixation during the auditory reward cue, and then once two discs had appeared, move their eyes as fast as possible to the *second* disc that was lit. The first onset thus acted as a distractor. It was explained to participants that the target would remain on the screen until they looked at it, but if an erroneous eye movement to the distractor was made, they would be delayed and thus win less. **C)** Examples of eye movements. Error trials were those on which the first saccade was towards the distractor (‘oculomotor capture’). **B)** After gaze arrived at the target, subjects were rewarded according to reaction time. Reward was calculated as a fraction of the maximum available (determined by each trial's incentive cue), using an exponential falloff. The falloff was determined adaptively using quantiles of the last 20 trials, in order to maintain a constant average reward rate over the course of the experiment.

Participants made speeded saccades to a target, while avoiding an early onset distractor, to win a reward. At trial onset, one of three discs was brightly lit, and participants were required to fixate this for 500 msec (Fig. 2A). They then heard a recording of a voice indicating the incentive on offer for that trial. Three reward levels were used: 0p, 10p or 50p (1 penny ≈ 1.5 US cents). This indicated the maximum amount participants could win on a trial, if they shifted their gaze rapidly to the target. Simultaneous with the voice, the fixation disc changed colour to yellow. Then, it was dimmed and the other two discs were illuminated in turn, with 80 msec interval between the two. The earlier onset disc was the distractor, and the later disc was the saccade target.

Participants were required to move their eyes as fast as possible to the target to win a fraction of the incentive on offer for that trial. After gaze arrived at the target, a numeric reward was displayed, proportional to the initial incentive cue but reduced depending on the time taken to reach the target. Eye movements were recorded and trials classified as an error or correct (Fig. 2B). Error trials were those on which an initial saccade was made to the distractor – so-called ‘oculomotor capture’ (Theeuwes et al., 1998). Participants were told that they would be paid in proportion to their performance at the end. Unknown to participants, adaptive criteria were used to maintain constant reward rates across participants (see below). This ensured comparable environments of reward probability for subjects who have different baseline velocities, or who fatigue over the duration of the experiment.

A PC running Matlab (The MathWorks) plus Psychophysics Toolbox was used to present stimuli on a CRT (1024 × 768 pixels; 100 Hz). A frame-mounted Eyelink 1000 (SR Research) infrared tracker monitored left eye position relative to the screen, sampled at 1 kHz. Eye movements were parsed online to provide trial-by-trial feedback. Participants sat 60 cm from the 21″ display against forehead- and chin-rest. Three screen locations were always indicated by dim grey discs, each 4° diameter, arranged in an equilateral triangle 11.4° apart. A non-ageing foreperiod of 1200–1600 msec separated the auditory cue and the distractor onset.

After the target appeared, the display remained until gaze arrived at the target. The time taken to reach the target (from distractor onset until gaze arrived at the target) was used to calculate reward (Fig. 2C) as follows:

Reward(t)=Rmax·min(eτ2−1τ1,1)

to the nearest penny, where *R* is reward for the current trial, *t* is the time taken to reach the target, *R*_max_ is the incentive value that could be won on a given trial, and *τ*_1_ and *τ*_2_ are adaptive reward criteria (see below).

As soon as gaze reached the target, reward was displayed as a red integer in the target disc. This was accompanied by a bell sound when the reward was 10p or greater, or a ‘cash register’ sound when 30p or greater was won. Importantly, the target location was then used as the starting point for the next trial. Thus trials formed a continuous sequence of saccades, without participants having to return gaze to any central fixation point. The next trial's target was chosen randomly from the two other possible destinations (discs) so that, over the experiment, there were an equal number of targets at each location.

Unknown to participants, the RT criteria *τ*_1_ and *τ*_2_ were adaptively adjusted using the last 20 trials. The criteria tracked quantiles of the RT distribution, keeping 10% of trials faster than *τ*_1_ and 30% of trials slower than *τ*_2_. The adaptive schedule tracked of the RT distribution over the 20 most recent trials irrespective of trial type. This ensured that participants experienced the full range of outcomes irrespective of their baseline reaction speed. Participants performed four blocks of 54 trials each, with a 2 min break between blocks, thus the experiment lasted approximately 40 min. There were three reward cues of 0p, 10p, 50p, three possible starting locations, and two possible target locations relative to this starting location.

### 2.3 Voxel-based lesion symptom mapping (VLSM)

Three behavioural variables of interest were chosen for VLSM for each participant:

1. Effect of value on vigour was calculated as the gradient of the *peak saccade velocity* as a function of maximum reward value on offer.
2. Clinical *level of apathy* measured by total LARS score.
3. Clinical *level of depression* indexed by the HADS.

Lesion mapping yielded a continuous-valued smooth lesion mask for each subject. For each voxel, the regressor of interest was correlated with the amount of lesion in that voxel, to obtain a *t*-statistic. The *t*-statistics were tested by permutation. Randomly relabelling each patient yielded the null distribution of the maximum and minimum *t*-statistic across all voxels (Kimberg, Coslett, & Schwartz, 2007). Each voxel's *p*-value was taken as the proportion of the null distribution that lay outside the actual computed value of the *t*-statistic. Voxels were only included in the analysis if four or more patients had nonzero lesion values in that voxel. The final map was thresholded at *p* < .05 and smoothed over a 2 mm radius.

## 3 Results

### 3.1 Behaviour

**Fig. 3:**
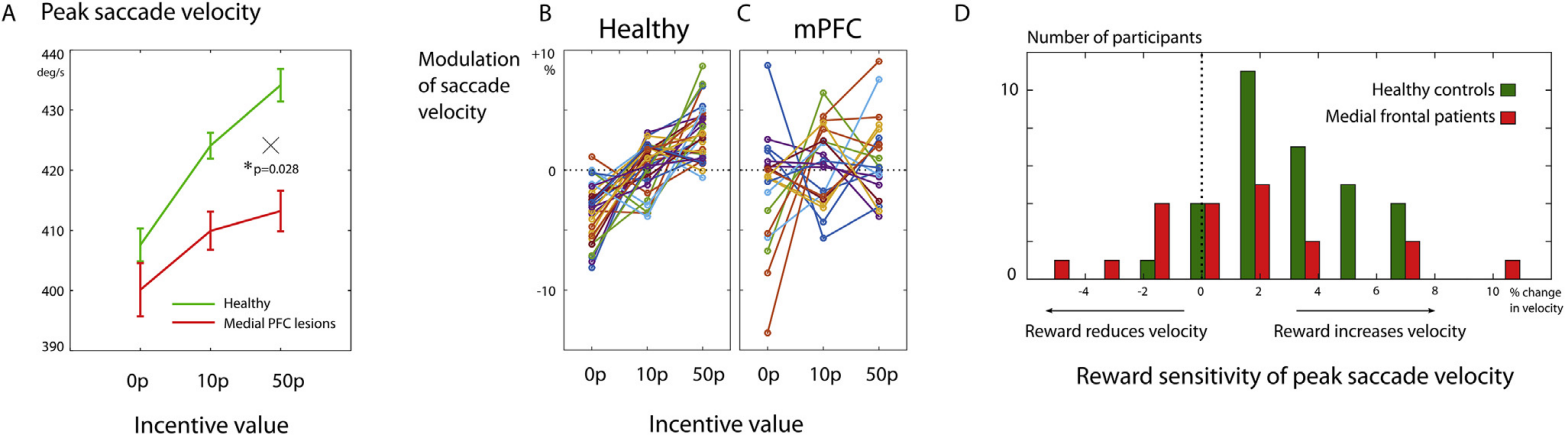
**Effects of reward value on saccade velocity. A)** The reward cue at the start of each trial influenced the subsequent saccade's velocity. Velocity was higher after a larger incentive cue. The effect is present in both controls (green) and patients with medial frontal lesions (red), but the patient group had smaller effects of value (group by reward interaction *p* = .028). Error bars indicate standard error of the mean within-subjects. **B)** Reward sensitivity data for each of 32 healthy volunteers. For each participant, the average peak velocity for each reward level is shown, relative to the overall mean velocity. **C)** Individual patients show heterogeneous effects of reward on saccade velocity (*N* = 19). Reward sensitivity of patients is considerably more variable than controls. **D)** The slope of reward sensitivity functions was calculated for each participant, and is depicted in a histogram. Note that several patients showed reduced reward sensitivity compared to controls, but a few patients had significantly higher effects.

We first examined how incentives influenced peak **saccade velocity** for eye movements that correctly landed on the target, and enquired whether there were differences between patients and controls. Overall, increased maximum reward value on offer was associated with increased saccade velocity [Fig. 3A, mixed effects general linear model (GLM) with reward value and group as factors, main effect of reward, *F*(1,105) = 37, *p* < .001]. The mean velocity was not significantly different between groups [*F*(1,102) = 1.21, *p* > .05], but importantly there was a significant interaction between group and reward [*F*(1,102) = 4.95, *p* < .05]. This indicates that patients did not increase their velocity in response to higher reward incentive cues as much as controls (Fig. 3A).

In **healthy control** participants, reward increased saccade velocity from 458 deg/s (mean ± s.e.m. 15) with zero incentive to 489 (±17) deg/sec with 50p incentive [mixed effects GLM with reward value, *F*(1,63) = 62, *p* < .001]. Saccade peak velocities are known to scale proportionally to movement *amplitude* (Bahill, Clark, & Stark, 1975), so it might be argued that these effects of reward on velocity actually relate to saccade amplitude. However, linear regression revealed that reward increased with velocity over and above that expected for a given amplitude (Fig. S1, effect of reward on residuals after accounting for amplitude, coefficient = 9.41 deg/sec per reward level ±1.32). Reward can therefore increase velocity independently, shifting the “main sequence” of saccades as recently reported in non-human primates (Chen, Hung, Quinet, & Kosek, 2013) and healthy humans (Manohar et al., 2015).

In the **patient group**, reward also increased saccade velocity [*F*(1,37) = 4.6, *p* < .05, from 443 ± 16 deg/sec to 459 ± 19 deg/sec]. Thus averaging across all patients, value effects on saccades were *not* abolished following medial frontal lesions.

However, considerable heterogeneity was noted within the lesion group (Fig. 3C and D). In particular, while at the group level patients had smaller effects of reward, depicted by shallower slopes on reward sensitivity plots, a few individuals showed *greater* sensitivity to reward than controls, manifest by steeper reward sensitivity gradients, with one patient 3 SD above the control mean (comparing individual patients to control distribution, *Z* = 3.41, *p* < .001).

Next, the effect of reward on **pupil diameter** was calculated at each time point after the onset of the auditory reward cue, using linear regression (Supplementary methods). In healthy volunteers pupillary dilatation, our measure of autonomic response, was greater when reward was high compared to when reward was low, on average increasing from −.08 (±.13, Z-scored units) with no reward to +.85 (±.12) after a 50p incentive cue [*F*(1,63) = 18, *p* < .001]. Patients also showed a trend to pupillary dilatation in response to higher reward cues [*F*(1,37) = 3.04, *p* = .06].

**Fig. 4:**
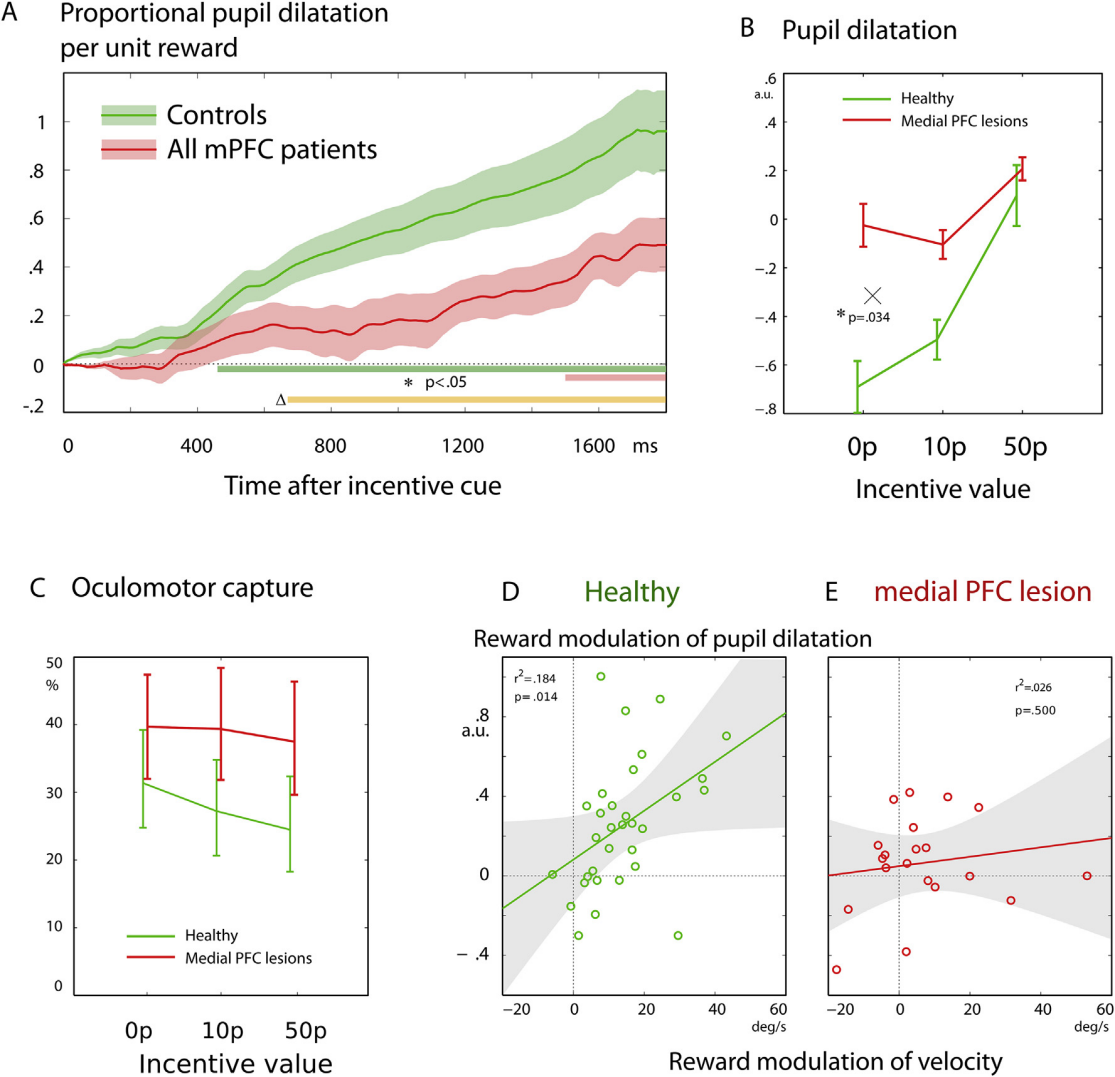
**Effects of reward on pupil dilatation and oculomotor capture. A)** After the auditory reward cue there was a 1200–1600 msec foreperiod. The effect of reward on the pupil size during this period is shown as a function of time after the cue. Values on the y-axis are the slope of the pupil-by-reward function, at each time point. A positive value indicates that at that moment, the pupil was larger on high-reward trials compared to low-reward trials—i.e., reward-related dilatation. Time points at which there was a significant effect of reward on pupil diameter are indicated by the green and red strip below (*p* < .05 using a linear model). In healthy volunteers, the pupil dilates in response to reward after 467 msec. Patients' pupils were not significantly influenced by reward until after 1400 msec. The difference between patients and controls becomes significant at 653 msec, indicating a smaller autonomic incentive effect before the target (group by reward interaction, yellow bar). **B)** The pupil diameter 1200 msec after the cue, relative to the pre-cue baseline, is shown as a function of reward size (proportional difference). Higher incentive values caused greater pupillary dilatation in healthy controls. This effect is diminished in patients (interaction *p* = .034). Error bars are standard error of the mean within subjects. **C)** The proportion of trials on which oculomotor capture errors (saccades to the distractor) occurred reduced as reward increased, suggesting increased motivational control over distraction. Patients and controls did not differ in the proportion of errors or in the effect of reward. **D** and **E**) The effect of value on velocity and pupillary dilatation was measured by the gradient of the lines in Fig. 3, Fig. 4B, for each individual. A scatter plot is shown for each individual's gradient of pupil dilatation at 1200 msec as a function of reward, and their gradient of velocity as a function of reward. Thus a high y-value indicates that a participant's pupil dilated strongly for high rewards compared to low rewards, whereas a high x-value indicates that their saccade velocity was much faster for high rewards compared to low rewards. **D)** In healthy controls, the effect of value on pupillary dilatation correlated positively with the effect of value on saccade velocity (*p* = .014), indicating that the same healthy participants who had greater pupil dilatation to rewards also increased their velocity more for reward. **E)** There was no such correlation among the patient group: the influence of reward on the pupil was independent of its effect on velocity, across patients.

Average curves are shown for patients and controls, demonstrating a reduced effect of reward after medial frontal lesions (Fig. 4A). Positive values indicate dilatation when the reward was high, with larger values indicating a steeper slope for the effect of reward. The difference between groups was first significant at 653 msec using a two-tailed two-sample *t*-test. The effect of incentive cues on the pupil dilatation at 1200 msec after the reward cue was compared using a linear model with group and reward as factors. As expected, there was a main effect of reward [Fig. 4B, *F*(1,102) = 13, *p* < .001]: higher incentive cues led to greater pupillary dilatation during the cue period. There was no main effect of group [*F*(1,102) = .30, *p* > .05], but there was a significant interaction between group and reward: patients had smaller effects of reward value on pupillary responses compared to controls [*F*(1,102) = 4.62, *p* < .05]. Thus, autonomic responses to reward value were also diminished in the lesion group (Fig. 4B).

**Error or oculomotor capture rates** were compared between groups, and as a function of reward value, using a linear model. Reward significantly reduced distractibility [Fig. 4C, main effect of reward, *F*(1,102) = 11.2, *p* < .001], but with no significant difference in distractibility between patients and controls [*F*(1,102) = 2.77, *p* > .05] and no interaction [*F*(1,102) = 2.11, *p* > .05]. This indicates that effects of valuation on distraction were similar in both groups. Reward effects on velocity did not correlate with reward effects on errors (Fig. S2), suggesting that our effects on saccade velocity could not be explained in terms of cognitive control or response inhibition. Thus reward modulation of cognitive control dissociated from modulation of movement vigour. The velocity of erroneous saccades was also higher with reward (Fig. S3).

### 3.2 Apathy and depression

Lille apathy rating scale (LARS) scores in patients ranged from −27 to −2, with 13 cases fulfilling criteria for clinical apathy (≥−16), of which five met criteria for severe clinical apathy (≥−9) (Table S1). Four had mild depressive symptoms (≥8 on the HADS), two had moderate depressive symptoms (≥12), and none had severe symptoms (≥14). There was no significant correlation between apathy and depression (Spearman *ρ* = .18, *p* > .4). Thus, in this sample, more patients exhibited apathy than depression. There were no significant correlations between apathy and behavioural reward sensitivity effects on velocity or pupil diameter, nor between the behavioural measures and depression (all *p* > .05).

The pronounced variability of the effects of lesions, across patients, led us to look for additional factors that might account for behavioural differences. To exclude the possibility that lesion size was a confounding factor, we measured total lesion volume (Table 1) but there was no correlation between *lesion volume* and reward effect on velocity (Spearman *ρ* = .019, *p* > .05), nor on overall mean velocity (*ρ* = −.17, *p* > .05) or clinical apathy scores (*ρ* = −.24, *p* > .05). Age was also excluded as a contributing factor to reward effects (*p* > .05). We therefore asked whether lesion location could be a causal factor.

### 3.3 Hypothesis-based analysis of effects of lesion location

Given the wide range of reward sensitivity observed (Fig. 3D), and heterogeneity of lesion locations *within* medial prefrontal cortex, we examined effect of location on reward sensitivity. We asked whether reward sensitivity, as measured by behavioural responses to the incentive value, correlated with lesions to a) regions reported to correlate with reward in fMRI studies; b) coordinates previously known to show aberrant activation in depression, and c) individual Brodmann areas (BA).

#### 3.3.1 Co-ordinates associated with reward response in fMRI studies of healthy people

**Fig. 5:**
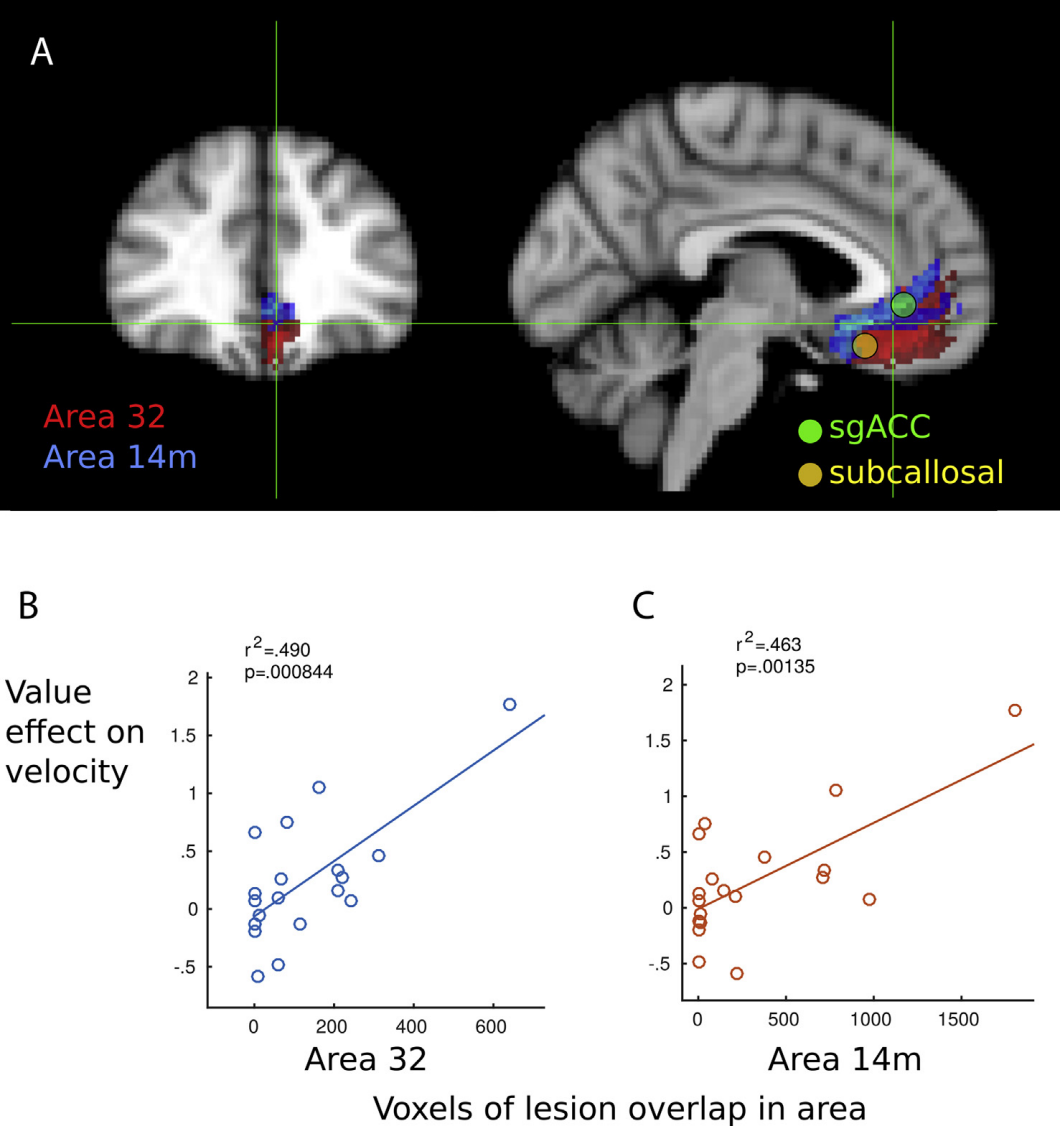
**Comparison with histological atlas. A)** The McGill atlas (Mackey & Petrides, 2014) gives five histologically defined regions in MNI space. Of these, two regions had more than four patients with damage, namely area 32 and area 14 m. These probabilistic maps are shown in blue and red respectively. Superimposed are the two vmPFC co-ordinates from the meta-analysis (Clithero & Rangel, 2014) that showed strong effects of reward in previous functional imaging studies. **B** and **C**) For the two histologically defined regions, area 32 and area 14 m, lesion maps were convolved with the probabilistic templates, to quantify the overlap of each patient's lesion with each region. Scatter graphs show the degree to which each of the 19 patients' lesions overlapped with area 32 and area 14 m template regions, and the corresponding individual's behavioural reward sensitivity, as measured by saccade velocity. Both regions showed significant positive correlations with reward sensitivity. Crosshairs show the centroid of the VLSM result of Fig. 6.

Co-ordinates from a recent meta-analysis of 81 imaging studies that localised value signals in medial PFC (Clithero & Rangel, 2014) were used to evaluate whether lesions at these locations affect reward valuation. In the meta-analysis, five peaks were reported. Of these coordinates, only subgenual ACC and subcallosal cortex had more than four patients with lesions in our study. We therefore asked whether value effects in our task were affected by lesions at these two locations (shown in Fig. 5A, green disc for subgenual ACC and yellow disc for subcallosal locations). A 5 mm-FWHM sphere around each coordinate was convolved with the lesion maps, to determine the *degree of lesion* for each patient, giving a value between 0 and 1 for that co-ordinate. This value therefore indicates the proportion of voxels within a 5 mm radius of the co-ordinate which are lesioned, for each patient, with zero indicating no lesions in the vicinity of the coordinate, and 1 indicating that the coordinate is deep within the lesioned area. Nine patients had lesions overlapping the subgenual ACC coordinate, and seven had lesions at the subcallosal coordinate.

The degree of lesion at the subgenual ACC coordinate (*x* = −2, *y* = 40, *z* = −4) correlated *positively* with the magnitude of *velocity sensitivity* to reward value (*r* = .59, *p* < .01). Thus patients with damage to this location increased their velocity in response to reward *more* than patients without damage to this region—i.e., their saccade velocity was increased more by reward. Lesions in subcallosal cortex (*x* = −2, *y* = 28, *z* = −18) also correlated positively with effects of reward value on velocity (*r* = .62, *p* < .01) Thus, lesions involving either of these two areas led to *increased* reward sensitivity, compared to lesions outside those areas, paradoxically conferring protection from the blunted reward effects seen at the group level. These correlations were robust to increasing the radius of the sphere to 10 mm, and to regressing out covariates age, digit span and lesion volume (Fig. S5). Lesions to subcallosal cortex were also associated with significantly reduced total *apathy* scores (Spearman's *ρ* = −.46, *p* < .05), with a similar trend at the subgenual ACC coordinates (*ρ* = -.42, *p* = .077). By contrast, depression as indexed by the HADS did not correlate with lesions at either location.

#### 3.3.2 Co-ordinates showing aberrant activity in depression

Coordinates 4 mm away from the subgenual ACC locus have previously been reported in a study that demonstrated altered vmPFC activity in depression (*x* = −2, *y* = 36, *z* = −4, Drevets et al. 1997). We examined the correlation with velocity reward effects as above, but using the coordinates associated with depression (Drevets et al. 1997). Damage to this location also showed trends to increase reward value sensitivity of saccade velocity (*ρ* = .42, *p* = .076), and correlated with clinical apathy scores (*ρ* = .47, *p* < .05), such that patients with damage at this locus were significantly *less apathetic* than patients with lesions elsewhere, echoing the analysis using the two regions identified by fMRI reported above.

#### 3.3.3 BA

Next we asked whether specific BA may be implicated in altering reward valuation. Each patient's lesion map was convolved with vmPFC regions defined in the McGill probabilistic histological atlas (Mackey & Petrides, 2014), to give the total volume of lesion intersecting the region. Five regions were examined: BA11m, 14m, 24, 25, 32. Lesions in area 32 and area 14 m both correlated *positively* with value effects on saccade velocity (Fig. 5B and C, *r*^2^ = .48, *p* < .001 and *r*^2^ = .45, *p* = .0018 respectively). Thus damage to these two areas was associated with larger reward-related increases in saccade velocity. There were no effects in areas 11 m, 24 or 25 (*p* > .05), although the lesion volume within each of these areas was low in our patient group, signifying low power to detect effects in those regions. Apathy as indexed by the LARS correlated *negatively* with lesions to areas 32 and 24 in the left hemisphere, such that lesions reduced apathy scores (*r* = −.68, *p* < .01 and *r* = −.48, *p* < .05 respectively). No correlations were found with the depression score. These findings corroborate the co-ordinate based analyses above, confirming that lesions to regions previously considered central to reward processing may indeed increase reward's effect on behaviour, and reduce apathy. The findings from this analysis are not independent of the previous coordinate-based analysis, due to some overlap between the hypothesis-based regions of interest (Table S2).

### 3.4 Voxel-wise lesion-effect mapping

How large is the region within medial PFC associated with altered reward valuation? To answer this, we next performed a whole-brain analysis, correlating the degree of lesion at each voxel with behaviour, to ascertain the volume over which the above correlations held. A voxel-wise analysis was used to examine whether the lesion amount at each voxel correlated with effects of reward on velocity, apathy and depression scores (see Methods). A map of the *t*-statistic for the ordinary least-squares regression for each voxel was constructed. To test for significance correcting for family-wise error, the *t*-statistic was calculated when the subjects' lesions were permuted 5000 times, and the probability of a significant result across the brain was used to threshold the map at *p* < .05, with threshold-free cluster enhancement using the FMRIB software library (FSL, Smith et al., 2004).

**Fig. 6:**
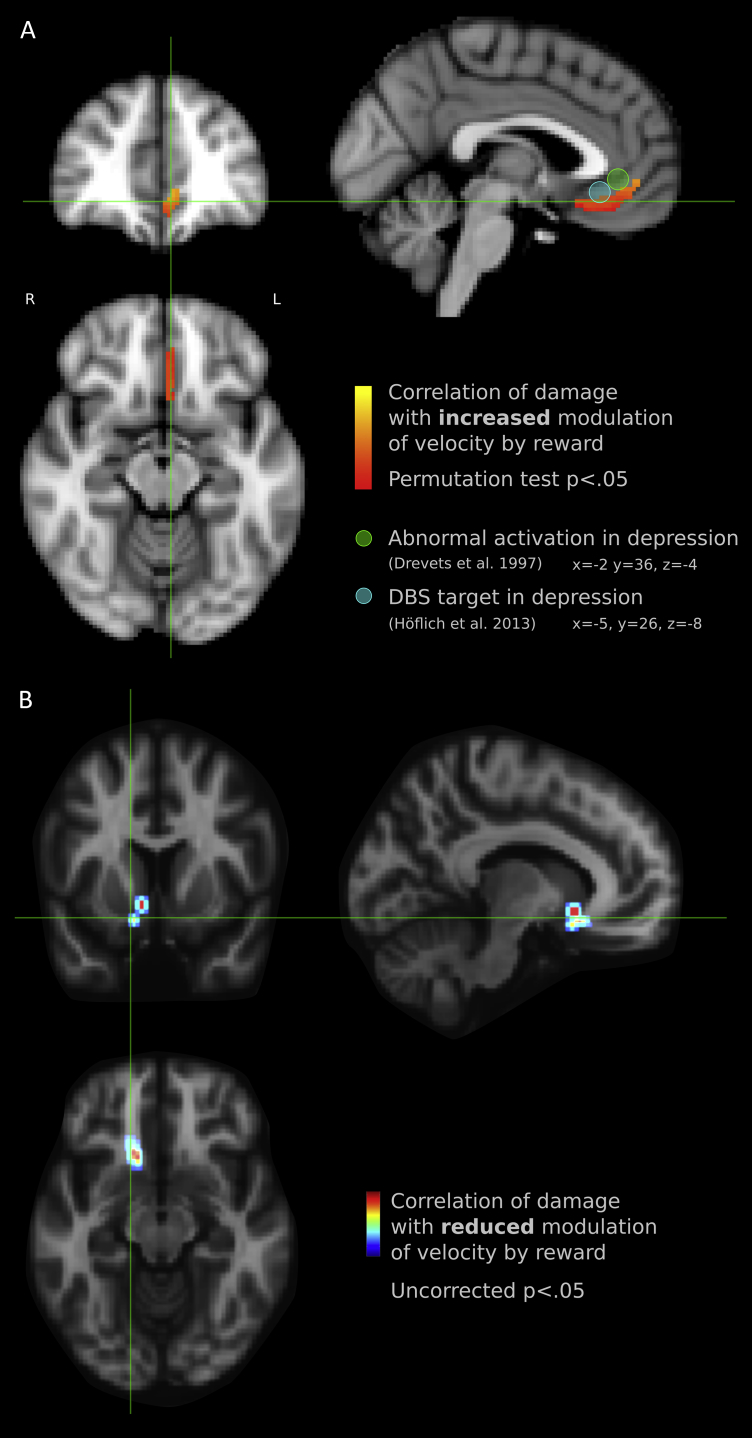
**Voxel-wise lesion-behaviour mapping of the effect of reward value**. A whole-brain analysis was performed to find regions in which lesions were associated with altered sensitivity to reward value. The degree to which a patient had lesion in a given voxel was correlated with the strength of the effect of value on saccade velocity. **A)** Permutation testing in conjunction with threshold-free cluster enhancement demonstrated a region in vmPFC in which damage correlated with increased value effects (*p* < .05 false detection rate corrected). **B)** When examining voxels which when lesioned correlated with reduced reward sensitivity, there were no voxels that survived correction for multiple comparisons. Images show regions with uncorrected *p* < .05.

A single region was found, located in vmPFC (Fig. 6A), in which lesions correlated with *increased* effects of value on saccade velocity. This establishes that, in our sample, patients who had damage in this area had *greater effects of value* on saccade velocity than patients whose lesions did not include this area. For comparison, we superimposed the subgenual cingulate white matter locus where DBS has previously been shown to alleviate depression (depicted as a 5 mm blue sphere, Mayberg et al. 2005) and a locus at which abnormal activation has been reported in depressed individuals (green sphere, Drevets et al. 1997).

On this whole brain analysis there were no regions in which lesions led to *decreased* reward effects on velocity, despite the fact that as a group patients showed, overall, reduced reward sensitivity indexed by velocity response. Were there *any* regions that contributed more than others, to reduced reward sensitivity? One approach sometimes adopted in previous vmPFC lesion studies, given the paucity of such lesion cases, is to report results without correcting for multiple comparisons (Tsuchida et al., 2010), whilst cautioning of the possibility of false positives. The whole-brain permutation test used above is a stringent criterion, which reduces power to detect small effects. The uncorrected statistical map was examined for trends to correlation. At the more liberal threshold of *p* < .05, there were some voxels which, when lesioned, predicted *reduced* incentive effects on velocity (Fig. 6B). This region actually survived correction if a ‘mirrored’ analysis was used, i.e., when hemispheres were reflected on to a single hemisphere, assuming left-right symmetry (Fig. S4). It includes anteromedial parts of nucleus accumbens, and the white matter tract just inferior and medial to it. This notoriously intricate region includes the medial forebrain bundle, greater terminal islands, and the anterior perforated substance including the diagonal band, nucleus basalis of Meynert, and part of the ansa lenticularis (Mai et al., 1997, Morel, 2007). Thus the dopaminergic and cholinergic inputs to vmPFC, and main outputs of the ventral pallidum, may all be jeopardised by lesions to white matter at this locus.

**Fig. 7:**
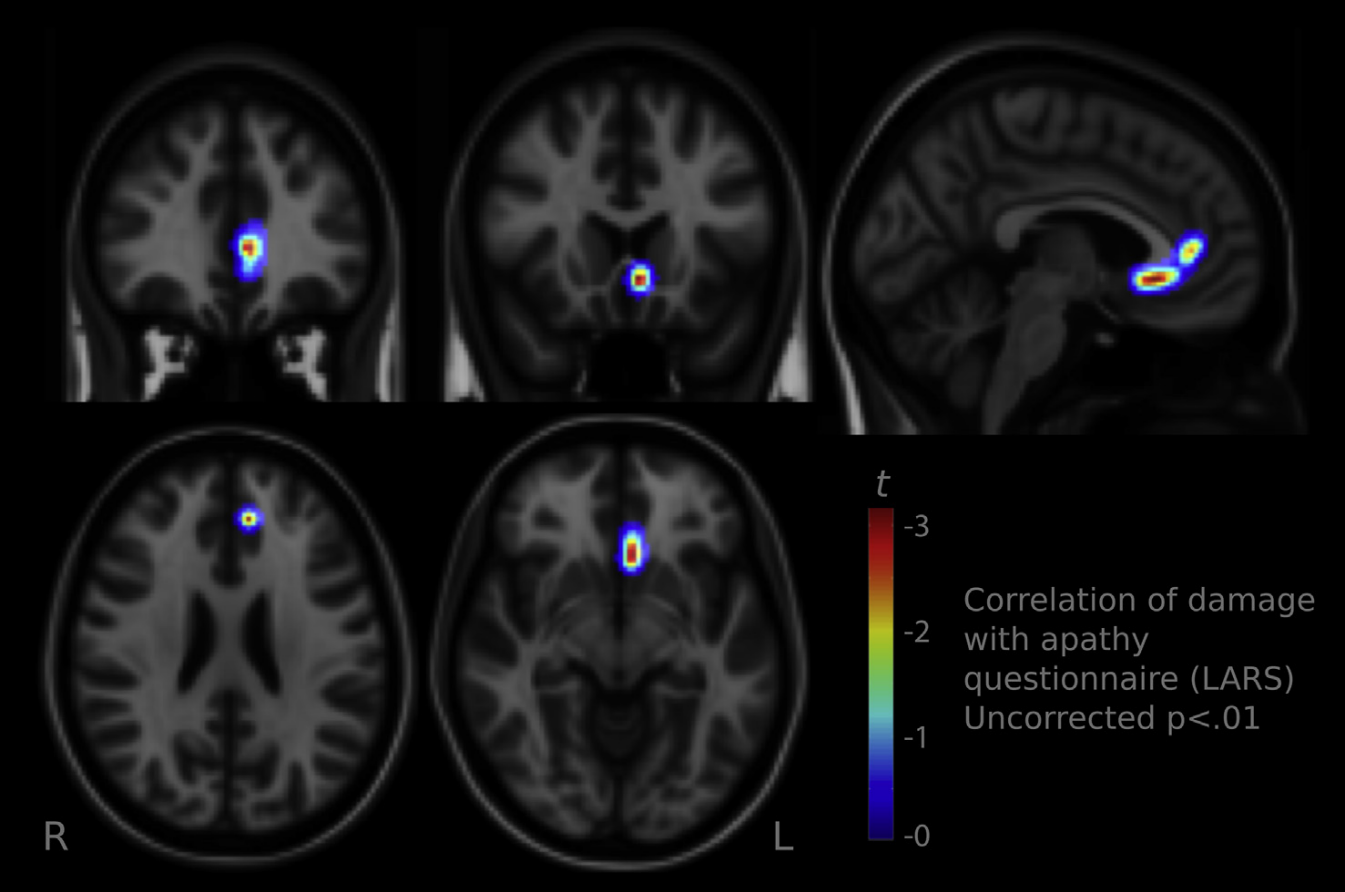
**Correlation of lesion location with clinical apathy**. The total score on the LARS apathy questionnaire correlated negatively with the degree of lesion in subcallosal cortex (co-ordinates shown in yellow in Fig. 5). To show graphically the extent of the area, the uncorrected correlation map is shown, thresholded at *p* < .01 for each voxel. This suggests that damage in this area was associated with *lower apathy* scores.

Finally we performed VLSM to find regions which correlated with *clinical apathy*, as measured by the LARS. No voxels remained significant when whole-brain correction for multiple comparisons was applied. Given that a significant correlation was found in the hypothesis-based ROI analyses above, we performed an exploratory analysis using an uncorrected threshold at *p* < .01. The map demonstrated a region in subgenual and pregenual ACC which, when damaged, leads to less apathy, compared to damage to other regions in the sample tested here (Fig. 7). Finally, to check there were no effects of lesions on baseline performance, we ran comparable correlations of the overall mean velocity (across all reward levels). There were no areas in which damage significantly correlated with baseline velocity, suggesting the associations with increased reward sensitivity of saccade velocity were not due to *generalised* changes in velocity.

Total reward attained was lower in patients during the initial blocks of the experiment, due to the time taken to adapt the RT criteria (Figs. S8 and S9). The velocity sensitivity analyses were repeated using only using later trials, in which there was no group difference in winnings. This analysis in fact strengthened the results (Fig. S10). This suggests that reward rate could not explain the differences in invigoration by reward in our data.

Because previous data on this task has shown reward most strongly affects velocity rather than distraction, and since no behavioural group differences were observed in oculomotor capture, the above analyses focused on velocity modulation by reward. Supplementary analyses demonstrated that reward modulation of distraction and velocity did not correlate (Fig. S2), and that there was no localisable lesion contribution to saccade errors or their reward sensitivity.

## 4 Discussion

This study enquired whether damage to medial PFC influences how behaviour was affected by incentives in a cohort of nineteen individuals with damage to the medial frontal lobe (Fig. 1). Using a simple, speeded saccade task with a monetary incentive pre-cue (Manohar et al., 2015), we measured invigoration by reward in terms of saccade velocity, pupil dilatation and distractibility (Fig. 2). In healthy people, incentives systematically increased saccade velocity and pupillary dilatation (Fig. 3). Overall, modulation of velocity and pupil dilatation by reward value was significantly *reduced* in the medial frontal patient group (Fig. 3, Fig. 4A). However there was considerable heterogeneity with respect to how incentives influenced behaviour. We therefore examined how variability in lesion location within medial frontal cortex affected outcome. Unusually for lesion studies, some cases showed *increased* sensitivity to reward as indexed by vigour of response – saccadic velocity (Fig. 3C). When correlations were performed between the degree of lesion at specific loci which have previously been determined in previous studies to be important in reward processing and in depression (Clithero and Rangel, 2014, Drevets et al., 2008), there was a positive correlation between lesions to these specific vmPFC locations and the effect of reward value on behaviour (Fig. 5).

These findings demonstrate that damage to some parts of vmPFC paradoxically confers protection from the blunted reward sensitivity observed in the group at large. When behaviour was related to damage to specific architectonic subregions, lesions to BA 14m and 32 were significantly correlated with *increased sensitivity* to reward (Fig. 5), which was then confirmed by whole brain voxel-wise analysis, surviving correction for multiple comparisons (Fig. 6A). There were no specific regions associated with *lower sensitivity* to reward which survived such correction, but uncorrected analysis suggests that these may include nucleus accumbens and associated medial white matter (Fig. 6B).

These findings may fit well with recent portrayals of vmPFC as playing a regulatory role in reward processing (Forbes et al., 2014, Mason et al., 2014), perhaps even censoring monetary incentive effects (Kirk, Harvey, & Montague, 2011) or inhibiting incentive drives (Man, Clarke, & Roberts, 2009).

Previous studies of patients with damage to vmPFC have revealed complex cognitive deficits which do not necessarily immediately suggest an inability to represent reward value. Lesions here have been reported to lead to a host of deficits: changes in personality (Eslinger and Damasio, 1985, Rolls et al., 1994), apathy (Jouvent et al., 2011, Lhermitte, 1986), impulsivity (Berlin et al., 2004), blunted affect (Angrilli et al., 1999), theory of mind deficits (Leopold et al., 2012), inability to detect unfairness (Gu et al., 2015), emotional lability (Beer et al., 2003, Ghaffar et al., 2008, Kim and Choi-Kwon, 2000) and poor emotion recognition (Rolls, Hornak, Wade, & McGrath, 1994), as well as altered disgust (Ciaramelli, Muccioli, Làdavas, & di Pellegrino, 2007) and brand biases (Koenigs & Tranel, 2008). In addition, while some studies have found a predisposition to depression (Kim and Choi-Kwon, 2000, MacFall et al., 2001), others have reported protection from the syndrome (Ellenbogen et al., 2005, Koenigs and Grafman, 2009, Koenigs et al., 2008).

Perhaps more compelling from the perspective of reward processing are reports of altered reversal learning of stimulus-reward associations (Fellows and Farah, 2003, Hornak et al., 2004, Tsuchida et al., 2010) and disturbed decision-making following vmPFC lesions (Fellows and Farah, 2005, Gansler et al., 2011, Gläscher et al., 2012, Levens et al., 2014; though see Manes et al., 2002) and higher betting in risk-related decisions (Clark et al., 2008, Studer et al., 2015). In addition some patients show altered information sampling (Fellows, 2006) and self-inconsistent preferences (Fellows & Farah, 2007). These manifestations might indeed be consequences of a more pervasive disorder of *reward evaluation*. But surprisingly, to the best of our knowledge, there has been no previous report that has directly examined the effect of vmPFC lesions on reward sensitivity in humans. In the present study, we tested only positively-valenced reward effects, thus it was not possible to determine whether motivation by penalties might also be altered by lesions, which would suggest a generalised change in processing motivational salience (Kahnt, Park, Haynes, & Tobler, 2014).

In our study, the results of hypothesis-led investigation of regions implicated in reward sensitivity from fMRI studies, and from neuroimaging studies of people with depression, revealed that lesions to specific areas of vmPFC appeared to reduce the likelihood of apathy. Moreover, VLSM using an uncorrected threshold at *p* < .01 demonstrated a region in subgenual and pregenual ACC which, when damaged, leads to less apathy, compared to damage to other regions in the sample tested here (Fig. 7). If confirmed in other samples, the findings on apathy together with those of *increased* reward sensitivity associated with damage to a similar area (Fig. 5, Fig. 6) suggest that there might be a relationship between protection from apathy and reward sensitivity.

Disorders of motivation seen in apathy and depression may involve at least two factors: on the one hand, diminished reward representations, and on the other, an augmented attenuation or regulation of incentive drive. These two factors might plausibly arise from interactions between ventral striatum and inferior frontal cortex (Der-Avakian and Markou, 2012, Jo and Mizumori, 2015). Such a fine-grained and nuanced system might partly explain the diversity of sequelae of medial frontal lesions.

No significant effects, either on the basis of lesion or behavioural analysis, were identified for depression scores. This might be due to the fact that in our sample there were more apathetic individuals than depressed cases, or that our index of depression (HADS) might not be as sensitive as our measure of apathy (LARS). Another possibility is that the relationship that has been reported between vmPFC and depression (Drevets et al., 1997, Hamani et al., 2009) might relate to the role of this region in motivating behaviour. Emerging evidence suggests that anhedonia, one component of depression, might in fact comprise a disorder of motivation (Treadway & Zald, 2011). Reduced reward sensitivity and/or disconnection of such regions from brain areas involved in action preparation might also underlie behavioural apathy (Adam et al., 2013, Martínez-Horta et al., 2014, Rochat et al., 2013).

The findings reported here show that both *increased and decreased* reward sensitivity can occur following medial frontal damage in humans, but there might be specificity to these effects depending upon lesion location. These results are not consistent with an account of vmPFC being involved in simply responding to reward cues. Single neuron studies in primates demonstrate cells in orbitofrontal cortex (OFC) which specifically respond to reward anticipation (Padoa-Schioppa and Assad, 2006, Tremblay and Schultz, 1999) or reward value in a context-specific manner (Rolls, 2000). Importantly, some neurons encode reward value positively, whereas others encode it negatively (Kennerley et al., 2011, Wallis and Miller, 2003), a finding mirrored in some neuroimaging studies (Metereau and Dreher, 2015, Plassmann et al., 2010). This makes it relatively difficult to predict how damage would affect reward processing. Predictions are further hampered by the many fine grained anatomical distinctions in this area, both functional and architectonic (Mackey and Petrides, 2014, Rudebeck and Murray, 2011).

One possibility is that expected rewards are in fact calculated in the basal ganglia, and these values are autonomously used to incentivise behaviour, but vmPFC receives these signals and is able to *modulate or contextualise value*, in order to regulate incentive effects (Niv, 2007, Wilson et al., 2014). The findings presented in our human lesion study would support theories in which ventral striatum may mount a primary response to reward, with vmPFC playing a more regulatory or evaluatory role (Der-Avakian & Markou, 2012).

In addition to directly observing behavioural motivation, we used pupil dilatation to index processing of rewards. Overall, patients had reduced pupillary dilatation in response to rewards (Fig. 4A). Functional imaging studies have suggested that autonomic arousal may be subject to regulation by dorsal medial frontal areas (Critchley, Tang, Glaser, Butterworth, & Dolan, 2005), whereas more ventral areas have been implicated in non-human primates (Reekie et al., 2008, Rudebeck et al., 2014). The changes we observed were not attributable to mood (Drevets et al., 2008).

One caveat to the findings reported here is that although the sample size was sufficient to determine some significant lesion specific effects, just as for any investigation of this nature, lack of positive findings cannot be taken to mean that they do not exist. Although we have demonstrated regions which when lesioned alter reward processing, we would not argue that vmPFC is the only brain area with such effects. Due to the number of patients, there was sufficient power only in vmPFC and pregenual ACC to detect small effects (80% power for 1 SD effect size). Therefore, it is not possible to draw conclusions as to whether any *other* medial frontal regions might also be involved. Recent lesion mapping studies have also shown how precise localisation using such techniques might in principle be difficult given that the vascular supply means that the each voxel is not necessarily independent of other nearby voxels in terms of probability of being damaged (Mah, Husain, Rees, & Nachev, 2014). However, the study of selected patients with relatively small lesions mitigates against these issues and, in addition, the investigation reported here was led by a specific hypothesis: to determine if lesions to vmPFC increase or decrease reward sensitivity.

The reward sensitivity changes we observed are not attributable to differences in distractibility, with no significant group differences in saccadic error metrics. We note that in our task, the distractor was salient but also task-relevant, thus combining elements of the oculomotor capture and antisaccade tasks. Thus this result suggests that neither salience processing nor cognitive control were directly affected by ventromedial lesions.

Thus deficits in the incentivisation of movement vigour were not, in our data, accompanied by altered motivational effects in cognitive control. This could simply be due to our binary measure of distractibility being less sensitive, compared to the velocity measures, such that small global changes in reward sensitivity were not reflected in error rates. However an alternative intriguing interpretation is that incentive modulation of movement vigour dissociates from modulation of cognitive control (Fig. S2), which perhaps requires integrity of more dorsal brain regions.

In conclusion, lesions to medial prefrontal areas have variable effects on reward processing. In terms of incentivisation of movement speed, damage to medial frontal cortex can reduce reward sensitivity whereas paradoxically lesions to some specific areas can increase reward sensitivity. This study provides causal evidence for a role of vmPFC in the evaluation of rewards. Disruption of this process may alter the normal regulation of motivational responses to incentives, leading to diverse behavioural syndromes observed in the clinic.

## Figures and Tables

**Table 1:** **Demographics of the patient group**. None of the patients had damage outside the ACA vascular territory. All had normal neurological examination except patient 1 who had downbeat nystagmus. LARS = Lille apathy rating scale; A/D = Hospital anxiety/depression score; LV = lesion volume in cm^3^. Breakdown of apathy subscales is given in Table S1.

	Age	Lars	A	D	LV	Centroid xyz			Lesion
1	46	−16	5	7	6.2	44.4	50.5	48.4	Bilateral dorsal ACC/SMA
2	44	−13	4	4	3.3	43.5	50.1	44.0	Left dorsal ACC + Right pregenual ACC
3	45	−16	12	3	16.6	44.0	43.6	40.5	Right dorsal ACC + bilateral pregenual ACC
4	61	−18	7	9	8.5	47.9	42.4	50.5	Right dorsal + pregenual ACC extending to PCC
5	63	−7	8	11	.1	37.4	45.7	40.5	Right dorsal ACC
6	56	−13	9	6	1.7	26.5	37.7	50.2	Left pregenual + subgenual ACC
7	61	−10	5	7	1.2	51.8	42.3	63.5	Left pregenual + subgenual ACC
8	57	−27	1	2	.9	74.4	78.5	83.3	Left pregenual + subgenual ACC
9	28	−22	6	5	6.5	75.3	83.8	81.2	Left medial OFC and pregenual ACC
10	48	−4			2.1	77.4	78.2	82.3	Left medial OFC
11	46	−16	3	7	9.1	78.6	81.9	91.5	Right medial OFC
12	45	−19	6	8	19.1	88.2	86.3	68.1	Right medial OFC
13	55	−5			2.6	90.1	80.4	77.0	Left anterior mOFC + medial frontopolar
14	33	−18	9	2	3.9	75.8	68.0	53.9	Bilateral mOFC + medial frontopolar
15	43	−10	7	7	11.5	54.0	32.2	48.7	Left medial OFC + medial frontopolar
16	70	−17	15	10	11.6	36.5	31.4	35.7	Left gyrus rectus + medial frontopolar
17	32	−13	9	6	1.9	29.7	33.3	56.4	Right gyrus rectus
18	58	−2	3	7	1.3	32.9	31.7	34.2	Right gyrus rectus
19	49	−19	11	9	.5	31.7	34.3	37.1	Bilateral posterior medial OFC

OFC: orbitofrontal cortex; mOFC: medial OFC.
