# Supplementary material for: Human ventromedial prefrontal lesions alter incentivisation by reward
Source: Cortex. 2016 Mar;76:104–20. doi: 10.1016/j.cortex.2016.01.005 (PMC4786053; doi:10.1016/j.cortex.2016.01.005)
Supplement: Supplementary file 1 [file mmc1.doc]

# Supplementary Materials

**Table S1: Apathy scores of patients, using the Lille Apathy Rating Scale (LARS, Sockeel et al. 2006)**

| Patient | Total | Produc-  tivity | Interests | Initiative | Novelty | Motiv-  ation | Emotion | Concern | Social | Self-awareness |
| --- | --- | --- | --- | --- | --- | --- | --- | --- | --- | --- |
| 1 | -16 | -3 | -2 | -3 | 0 | -3 | -2 | -4 | -1 | 2 |
| 2 | -13 | -4 | -1 | -2 | -4 | 2 | -1 | -2 | -2 | 1 |
| 3 | -16 | -4 | 1 | -3 | -3 | -1 | -3 | -2 | 2 | -3 |
| 4 | -18 | -4 | -2 | -1 | -3 | -2 | 0 | -2 | -4 | 0 |
| 5 | -7 | -2 | -2 | -2 | 0 | 0 | -2 | -1 | 4 | -2 |
| 6 | -13 | -4 | 0 | -3 | 0 | -2 | 2 | -1 | -2 | -3 |
| 7 | -10 | -4 | -1 | -4 | 3 | 2 | -2 | 2 | -4 | -2 |
| 8 | -27 | -2 | -4 | -4 | -3 | -4 | -2 | -2 | -4 | -2 |
| 9 | -22 | -2 | -4 | -4 | -2 | -4 | -3 | 1 | -2 | -2 |
| 10 | -4 | -2 | 1 | -3 | 1 | 1 | 0 | -2 | 0 | 0 |
| 11 | -16 | -4 | -2 | -2 | -4 | 0 | -1 | 0 | -3 | 0 |
| 12 | -19 | -3 | -2 | -4 | 0 | 0 | -3 | -3 | -3 | -1 |
| 13 | -5 | 0 | -1 | 0 | -1 | 0 | -4 | 1 | 2 | -2 |
| 14 | -18 | -3 | -1 | -4 | -2 | -4 | -4 | 0 | -1 | 1 |
| 15 | -10 | 1 | -3 | -4 | 0 | -3 | 0 | 1 | -2 | 0 |
| 16 | -17 | -3 | 1 | -1 | -1 | -3 | -3 | -1 | -2 | -4 |
| 17 | -13 | -4 | -2 | -4 | -3 | -3 | 4 | 2 | -3 | 0 |
| 18 | -2 | -2 | 0 | -4 | -3 | 2 | 0 | 4 | 3 | -2 |
| 19 | -19 | -4 | -3 | -4 | -3 | 0 | 2 | 0 | -4 | -3 |

### Supplementary Methods

***Patient details***

A database of 453 consecutive patients admitted under the neurosurgical team with subarachnoid haemorrhage was screened. Of these patients, 120 had anterior communicating artery aneurysms (including pericallosal artery and A1/A2 segment) or were angiogram negative. The scans of these patients were reviewed, and 48 were found to have lesions in the medial prefrontal cortex. Thirty of these were alive and well, with grade 1 or 2 haemorrhage, and 22 were contactable and mobile.

Of the 19 patients who had usable eye movement data, 15 had returned to work, 2 were retired, and 2 had not gone back to work due to reduced memory and motivation. None of the patients had a past medical history of mental health problems or previous neurological illness. Three patients had metal aneurysm coils that produced mild fMRI artefacts but these were confined to approximately 5 mm radius from the anterior communicating artery, thus not encroaching on the traced lesion locations.

***Behavioural analysis***

Saccades were parsed using criteria on velocity of 30 deg s^-1^, acceleration > 8000 deg s^-2^ and amplitude > 0.15 deg. On each trial the response was classified as correct or a distraction error (“oculomotor capture”) according to gaze trajectory after initiation of the first saccade (**Fig. 3C**). Trials were classed as correct if the first saccade amplitude was greater than 5 degrees and its endpoint was closer to the target than the distractor. Trials with blinks (3% of all trials) and saccadic RTs over 1 second (6.9%) were discarded. Blinks were classified as any loss of eye position after distractor onset and before the first 5 degree saccade . RT was defined as the time from distractor onset to initiation of the first saccade larger than 5 deg.

For each correct saccade, peak saccade velocity was computed as an index of response vigour ( see (Manohar et al. 2015). The peak saccade velocity was calculated as the maximum value of the displacement in windows of 3 ms. Value effects were measured by taking the mean peak saccadic velocity at each reward level, and computing the slope as a function of reward. The *autonomic response* to the different values of reward cue was measured using pupillometry. On each trial, the change in pupil diameter was measured 1200 ms after the auditory cue, compared to pre-cue baseline. The mean pupil dilatation for each reward condition was used to calculate a slope, indicating reward sensitivity of the pupil. Distractibility was measured as the proportion of trials on which error responses were made, i.e. on which the first saccade was in the direction of the distractor (so-called ‘oculomotor capture’ Theeuwes et al. 1998).

Pupil diameter was recorded from the onset of the auditory reward cue, over the 1400 ms duration of the foreperiod. The diameter was subtracted from a baseline calculated for 500 ms prior to the cue, and divided by the baseline to give the proportional change in pupil size. Pupil diameter trace after the cue was smoothed over 100 ms time bins, interpolating over gaps shorter than 500 ms. At each time bin, a linear model was used to extract the dependence of pupil size on the cued maximum reward. Positive values indicate that the reward dilated the pupil, whereas negative values indicate constriction with reward (**Fig. 5A**). Saccade amplitude was calculated as the distance of the saccade endpoint from its starting point.

***Coordinate-based analysis with lesions reflected onto a single hemisphere***

Note that the subgenual ACC and subcallosal coordinates from the meta-analysis were in the left hemisphere. Since function of the medial frontal cortex in value processing is often taken to be bilateral, we repeated the analysis with the patients' lesions mirrored onto one hemisphere. This makes the assumption that functional localisation in these areas is bilateral. In this analysis the correlations with incentivisation of velocity remained significant, with the effect of value on velocity influenced by lesions to both ACC (*r*^2^=0.30, p=0.0136) and subcallosal cortex (*r*^2^=0.23, p=0.036). Voxel-based analysis was performed using the mirrored hemispheres, in the same manner as in the Methods, using permutation testing with threshold-free cluster enhancement (**Fig S1**). In this analysis, which increased the power to detect effects while assuming symmetry, there were regions in which damage correlated significantly both with increased and decreased sensitivity to reward.

# Supplementary Results

### 1. Reward breaks the main sequence of saccades

To demonstrate that reward alters the standard relation between amplitude and velocity of saccades, saccade velocity and amplitude is shown as a function of reward (**Fig S1A,B**). For each participant, the increase saccade velocity over and above the “main sequence” was calculated, using linear regression. This gives the velocity residuals after factoring out the effect of amplitude on velocity, (**Fig S1C**), showing modulation by reward. Further, a binned plot of velocity as a function of amplitude is shown (using a sliding window 20 percentiles in width) for the three levels of reward, averaged over all 32 control participants (**Fig S1D**). This demonstrates that, while larger saccades are faster, reward increases the speed for a given size.


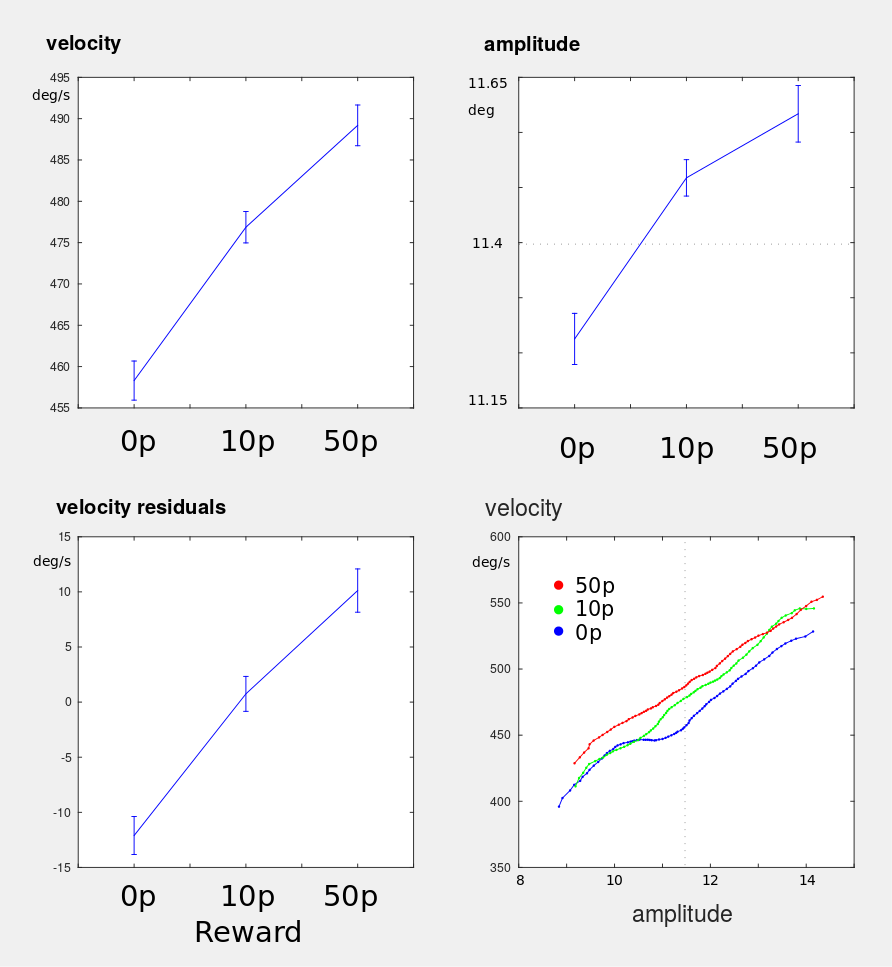


**Fig. S1 Effect of reward on saccade amplitude.**

A) Saccade velocity as a function of reward, for the healthy controls, as shown in the main text (for comparison)

B) Saccade amplitude is modulated by reward

C) Saccade velocity residuals were calculated from linear regression of velocity against amplitude, for each subject. The residuals are modulated by reward in both groups, indicating rewards increase velocity independently of amplitude. Error bars indicate within-subjects s.e.m.

D) Sliding bin plot showing the main sequence of saccades, and how it is modulated by incentive. Although saccade amplitudes are increased, peak saccade velocity increases more than expected given the amplitude increase. In other words, the main sequence in broken by reward.

### 2. Saccade errors

Although the focus of the study was on reward effects, our task was also designed to examine saccade errors. We also considered whether lesions might lead to increased distractibility. First, we note that our study is in agreement with other studies that find that reward modulates oculomotor capture. In patients, the proportion of oculomotor capture was 38% (±18% s.d.), and 30±16% in controls. This increase in distractibility was not significant (t(50)=1.66, p=0.10), and although the reward-slope for capture was also shallower in patients (2±8% compared to 6±10% in controls), this interaction was also not significant (t(50)=1.32, p=0.19). So although oculomotor capture is sensitive to reward, it is a) no greater in patients, and b) no less sensitive to reward in patients, who show do signs of reward insensitivity in other measures.

One possibility is that the mechanisms modulating cognitive control by reward are not dependent on medial frontal structures. However a simpler explanation could be the small effect size and nonlinearity of binary error-rate measures leads to reduced power to detect reward-insensitivity in distractibility.

In order to address whether oculomotor capture depended upon lesions, we two supplementary voxel-wise analyses of oculomotor capture rate. First we specifically asked whether damage to any medial area correlated with increased capture. This showed no significant regions after correction for multiple comparisons. Second, analogous to the main-text analysis of reward sensitivity for velocity, we used the *slope* of reward effect upon oculomotor capture rate as the regressor for voxel-wise analysis. This demonstrated no regions either reducing or increasing reward sensitivity for capture (both p>0.05).

Participants responded to reward in two ways: reducing capture, and increasing velocity, i.e. by improving both motor and cognitive control performance. We therefore asked whether there was a relationship between sensitivity to reward in these two measures – i.e. whether the slope of velocity reward effects correlated across subjects with the slope of capture effects. The reward effects did not correlate across these two domains (**Fig. S2**, *r*^2^=0.01, p>0.05). An individual’s improvement in capture with incentives did not predict their speed increase. This fits with our previous findings on this task, which we have taken to suggest separate motivational pathways governing motor vigour and cognitive control.

The absence of a significant effect of medial PFC lesions on oculomotor capture indicates that our findings of altered invigoration of velocity by reward cannot be explained in terms of changes in cognitive control. If lesions had in fact increased distraction, then it would be potentially more difficult to interpret our findings of reward sensitivity. Further, the findings are consistent with ventromedial frontal areas not being classically involved in distractor suppression.


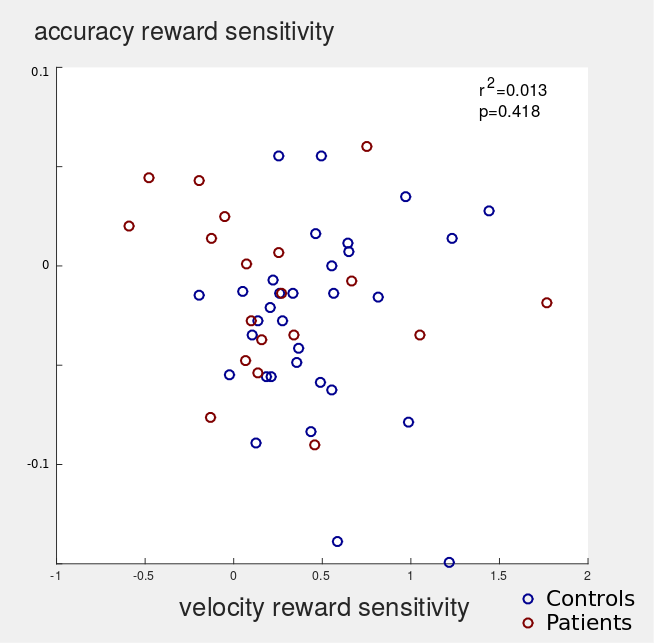


**Fig S2: No correlation between velocity sensitivity to reward and modulation of oculomotor capture by reward.**

For each participant, the slope of the reward sensitivity function was measured for peak saccade velocity, and pupillary dilatation in response to the cue. These two reward sensitivity measures did not correlate across individuals.

### 3. Error saccade velocities

For the primary analysis of saccade velocty, error saccades were excluded, as these saccade types are very different in their underlying mechanism with error saccades being driven by bottom-up processes and correct saccades by top-down processes. To examine whether these oculomotor capture saccades showed similar effects to correct saccades, a parallel analysis of error saccade velocity was performed (**Fig. S3**). This indicates that the peak velocity of error saccades did increase with reward (F(1,102)=6.11, p<0.05) but did not differ between groups (F(1,50)=0.48, p>0.05), just as for correct saccades. However there was no interaction between group and reward (F(1,102)=1.8, p>0.05).

Caution is required in interpretation of these null effects because a) individuals had widely varying proportions of oculomotor capture trials, resulting in a low power to detect effects on this metric, and b) capture saccades had generally shorter and more variable amplitudes compared to correct saccades.


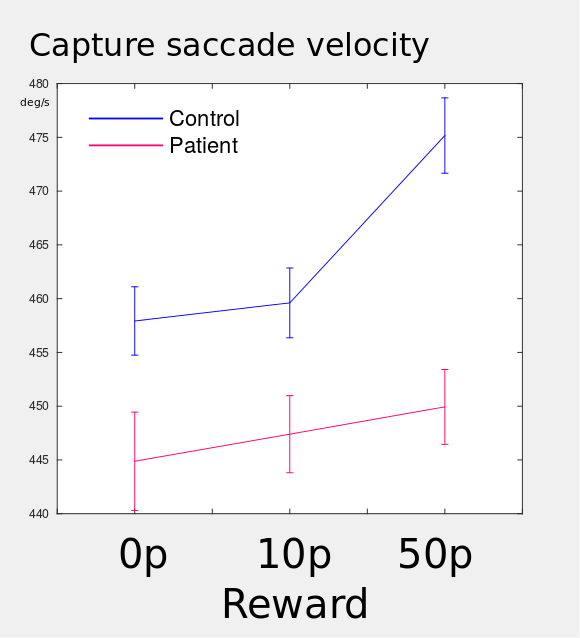


**Fig S3: Peak velocity of saccades which went to the distractor**

Reward increased velocity of captured saccades. However there was not significant difference between groups and no interaction between group and reward.

### 4. Regions correlating with reduced reward sensitivity, combining hemispheres

In the primary voxel-based analysis, we examined how reward sensitivity of saccade velocity correlates with damage to brain regions. No voxels correlated with *reduced* reward sensitivity afte correction for multiple comparisons, though a trend was observed in ventral striatal lesions. To examine this in more detail with greater power, the lesion map for each patient’s left and right hemisphere was reflected onto a single hemisphere. Voxel-based lesion analysis was performed on the combined hemispheres to give a single-hemisphere statistical map (**Fig. S4**). This was tested using permutation and threshold-free cluster enhancement just as for **Fig. 6**. For increases in reward sensitivity, the same region was significant as in the un-reflected analysis. For reductions in reward sensitivity, a region near nucleus accumbens did show significant effects, which in the un-reflected analysis did not survive multiple comparisons.


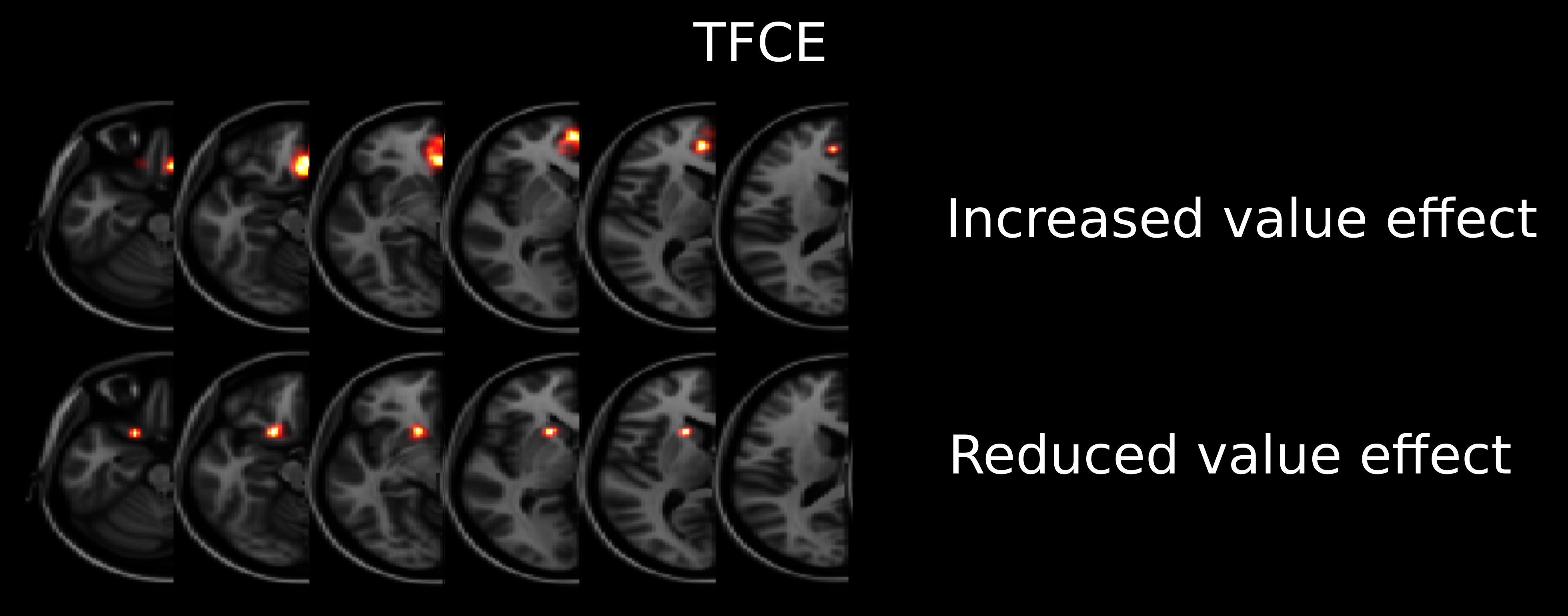


**Fig. S4: Voxels at which damage correlated with reduced velocity by reward**

Voxel-wise correlation when the lesions were collapsed across both hemispheres. Damage in the highlighted areas correlated significantly with reduced reward sensitivity, as measured in terms of saccade velocity modulation (Corrected for multiple comparisons using FSL permutation test with threshold-free cluster-enhancement p<0.05)

### 5. Results robust to covariates

Velocity sensitivity did not correlate with age or lesion size, so it is unlikely that these potentially confounding differences between individuals could explain our results. However to exclude this possibility, and demonstrate the robustness of our findings, these variables were factored out in the following three auxiliary analyses:

1. For our main coordinates-of-interest analysis of velocity sensitivity to reward, we included lesion volume and age as covariates of no interest. The result remained strongly significant, with t(18)=3.84, p<0.001 in subgenual ACC, t(18)=2.71, p=0.01 in subcallosal cortex, and t(18)=2.91, p<0.005 at the coordinates associated with depression.
2. We also had a measure of digit span on 17 out of 19 patients. This provides a coarse indication of cognitive ability. We included this measure (imputing the mean for the 2 patients without a working memory measure) in addition to lesion volume and age. This analysis gave comparably strong results: subgenual ACC t(18)=3.80, p<0.001, subcallosal cortex t(18)=2.58, p<0.01, depression-associated locus t(18)=2.79, p<0.01.
3. We added these three covariates in the voxel-based analysis of velocity sensitivity to reward. With these included in our analysis, the effect remained significant in essentially the same region when lesion volume, age and digit span were all included. The significant regions are shown in **Fig S5**.


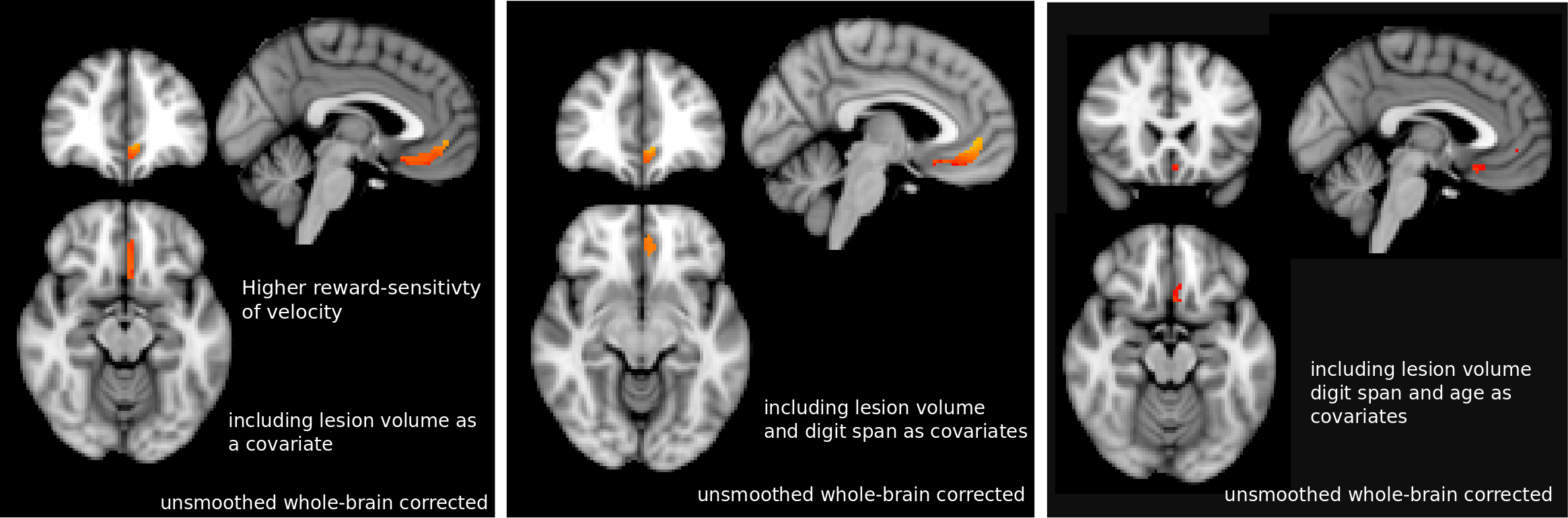


**Figure S5: Robustness of voxelwise analysis to covariates**

Voxel-wise correlation (as in main **Fig. 6**), covarying out effects of lesion volume, age and digit span, corrected for multiple comparisons using permutation testing with threshold-free cluster enhancement (FSL 5.0). All three analyses resulted in significant regions in vmPFC.

We note that when a single-voxel correction for multiple comparison was used, rather than a cluster threshold, then the effect was significant without covariates, and when volume was used, but not when the other covariates age and digit span were included.

### 6. Does pupil dilatation relate to lesion location?

To answer this question, pupil dilatation in response to reward was entered into the voxel-based analysis. After correcting for multiple comparisons over voxels, there were no significant areas which correlated with reward influence of pupil dilatation. The map of voxels that are significant at an uncorrected threshold of p<0.05 is shown (**Fig S6**).

Using the same region-of-interest analyses used for velocity, the degree of lesion to subgenual ACC (x=-2, y=40, z=-4) correlated positively with pupillary sensitivity to reward (r=0.62, p=0.005). Lesions to subcallosal cortex (x=-2, y=28, z=-18) showed only a trend (p=0.07).


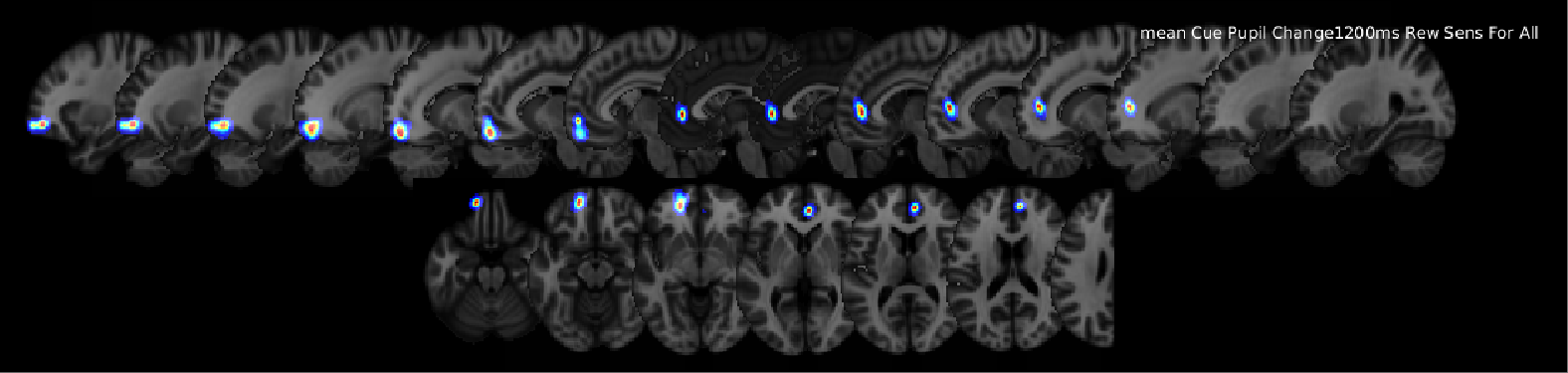


**Fig. S6: Effect of lesions on pupillary dilatation to reward**

Uncorrected map (voxelwise threshold p<0.05), indicating regions in which damage was correlated with increased pupil sensitivity to reward. These results appear to mirror the more robust velocity sensitivity changes demonstrated in the main text.

These brain-pupil relationships should be interpreted with caution. Firstly, due to the locations of lesions we had under 60% power to detect effects in the dorsal ACC / SMA. So a negative result there cannot be interpreted. Second, pupil effects correlated with velocity effects across individuals (**Fig S7A**). This suggests that autonomic value responses should not be thought of as independent of velocity effects. However there were significant correlations across trials in several individuals – sometimes showing faster saccades with dilated pupils, and others showing slower saccades (**Fig S7B**). This suggests there is a complex relationship between within-subject arousal effects, and inter-individual differences.


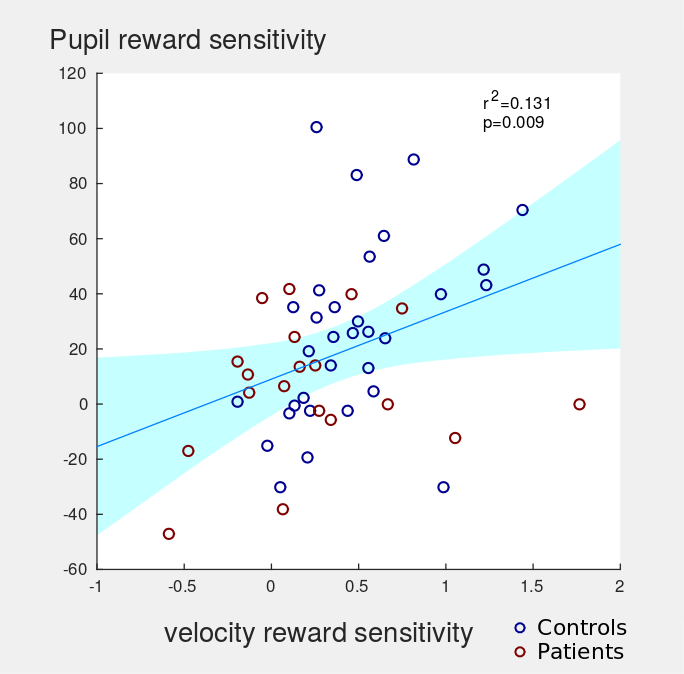

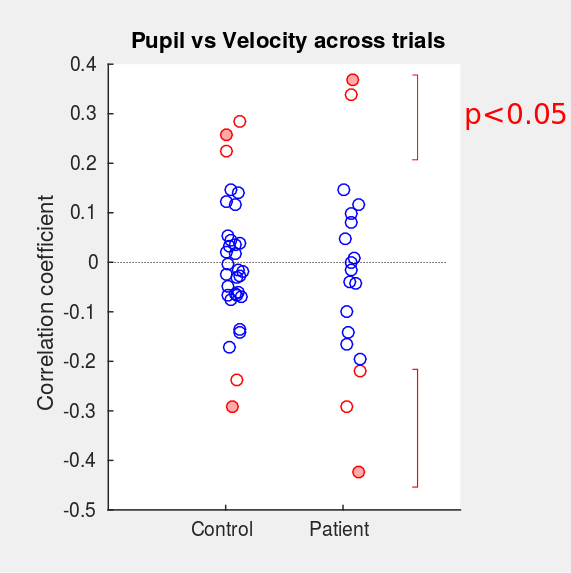


**Fig. S7: Reward sensitivity correlates across individuals between pupil diameter and saccade velocity**

a) Individuals who showed stronger modulation of velocity by reward, also showed stronger pupillary changes with reward. b) Within an individual, the pupil dilatation on a given trial in response to the reward cue could be predictive of the velocity of the upcoming saccade. The correlation coefficient, across trials, for each participant is shown. Points in red showed significant correlations (uncorrected Pearson’s p<0.05; filled points survived Bonferroni correction for 51 comparisons).

### 7. Total amount won did not explain the velocity sensitivity effects

Although our adaptive reward procedure was designed to balance average reward rate, this was based upon reaction times, and thus our algorithm does not guarantee identical overall rewards across individuals. Since RT criteria for earning reward were adjusted gradually with a 20-trial time constant, there remained a possibility of a difference between the groups’ rewards, because the adaptive reward needs to “learn” a subject’s speed at the start of the task.

We therefore examined the actual amount won. It turns out that patients in fact won less than healthy volunteers (**Fig. S8**). This was attributable to differences in the early part of the experiment, during which the adaptive reward schedule adapted to individual differences in RT.


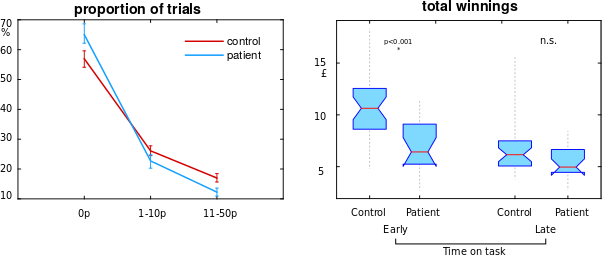


**Fig. S8: Patients obtained lower reward in earlier part of task**

A) Patients won less overall, with a smaller proportion of high-reward outcomes.

B) The difference between patients and controls was only evident in the first portion of the experiment. Trials were divided into “early” and “late” periods, and there was no significant difference between groups in rewards obtained later in the task.

We therefore split trials according to time on task, into two groups, “early” and “late”. The first half of the task showed group differences in total reward obtained (p<0.001). The second half of the task showed no difference (p>0.05). Accordingly, an ANOVA demonstrated a significant time x group interaction (F(1,67)=6.00, p<0.5).

In order to ensure that the reward was not the driver of reduced reward sensitivity in velocity, we used a similar early/late split on saccade velocity (**Fig S9**). A 3-way ANOVA examined effects of incentive (0/10/50p), time on task (early/late), and group (patients vs controls). As per the original analysis, there were significant effects of reward (F(1,249)=31.8, p<0.001). There was also a trend to interaction of reward by group (F(1,249)=3.45, p=0.064), in keeping with the significant interaction found in the primary analysis. However there was no effect of time, and no interactions between time x reward, time x group, or time x reward x group (F(1,249)=0.02, 0.46, 1.90 and 0.063 respectively, all p>0.05).


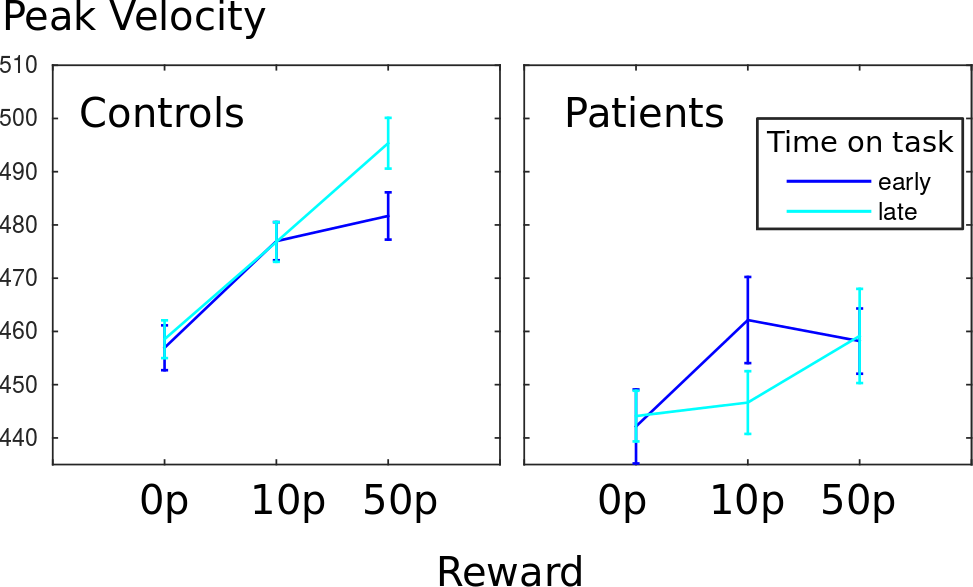


**Fig. S9 Reward sensitivity same in early and late part of experiment**

Since total reward obtained was not identical between groups in the early part of the task, we asked whether the velocity sensitivity differed between as a function of time on task. The task was split into two halves, with early and late trials analysed separately. There was no difference in velocity, or reward sensitivity, and no interaction with group.

Finally to show that the reward differences in the early phase did not influence our main result, we took reward sensitivity to be the slope of velocity effects observed in the later trials only. These slopes were then correlated with lesions using the same voxelwise analysis as in the primary result (Main text **fig. 6**). The results were highly similar (**Fig S10**), and were in fact slightly stronger than the original whole-task analysis, with minimum corrected p-values of 0.011, compared to p=0.018.

**
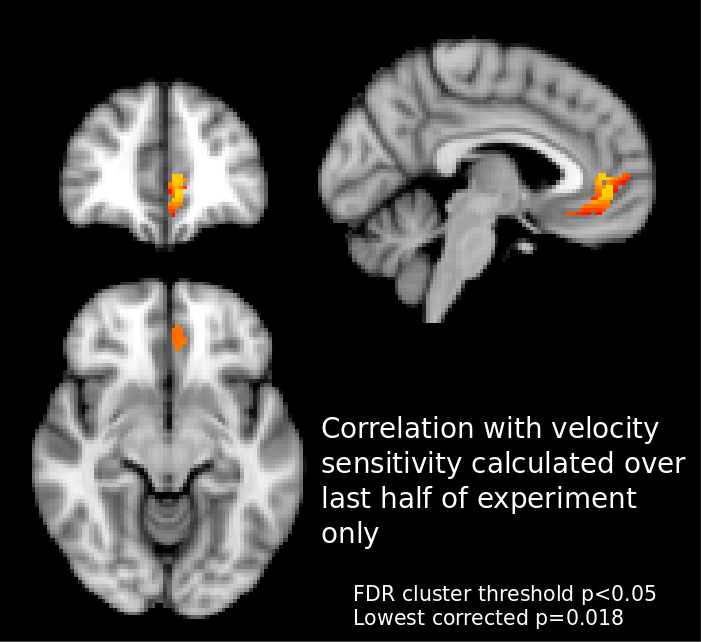
**

**Fig S10: Correlation of velocity with lesions, in second half of task**

The reward sensitivity slope calculated from peak saccade velocity in the second half of the task was used in voxel-based analysis. The analysis was performed identically to main **Fig. 6**, using whole-brain multiple comparison correction by threshold-free cluster enhancement. The effect was marginally stronger than in the main analysis.

### 8. Overlap between regions of interest

The regions of interest selected for the hypothesis-based analysis do have some overlap. To fully understand the differences in results between the comparisons presented, the number of voxels of overlap for each pair of regions is presented. A 5mm radius kernel was used around each of the three coordinate analyses, whereas the Brodmann areas were defined probabilistically from the McGill atlas. Since the regions are smooth-edged, we show voxel counts for overlap at a threshold of 10%.

|  | Clithero & Rangel | | Drevets | Mackey and Petrides | |
| --- | --- | --- | --- | --- | --- |
|  | Subgenual  ACC | Sub-callosal | Depression | BA32 | BA14m |
| Subgenual ACC | 57 | 0 | 23 | 33 | 51 |
| Sub-callosal |  | 57 | 0 | 48 | 1 |
| Depression |  |  | 57 | 21 | 56 |
| BA32 |  |  |  | 951 | 226 |
| BA14m |  |  |  |  | 595 |

**Table S2: Overlap between regions of interest reported.**

Diagonal values indicate total volume of the regions of interest, in 2mm voxels. Overlap is indicated in the off-diagonal elements. Subgenual ACC lies almost entirely within area 14m, but does not overlap with the subcallosal cortex region of interest. Note that the Brodmann areas as defined in the McGill atlas are much larger than the coordinate-based regions.
